# Selenoproteins Are Essential for Proper Keratinocyte Function and Skin Development

**DOI:** 10.1371/journal.pone.0012249

**Published:** 2010-08-18

**Authors:** Aniruddha Sengupta, Ulrike F. Lichti, Bradley A. Carlson, Andrew O. Ryscavage, Vadim N. Gladyshev, Stuart H. Yuspa, Dolph L. Hatfield

**Affiliations:** 1 Molecular Biology of Selenium Section, Laboratory of Cancer Prevention, National Cancer Institute, National Institutes of Health, Bethesda, Maryland, United States of America; 2 Laboratory of Cancer Biology and Genetics, National Cancer Institute, National Institutes of Health, Bethesda, Maryland, United States of America; 3 Brigham and Women's Hospital and Harvard Medical School, Boston, Massachusetts, United States of America; Auburn University, United States of America

## Abstract

Dietary selenium is known to protect skin against UV-induced damage and cancer and its topical application improves skin surface parameters in humans, while selenium deficiency compromises protective antioxidant enzymes in skin. Furthermore, skin and hair abnormalities in humans and rodents may be caused by selenium deficiency, which are overcome by dietary selenium supplementation. Most important biological functions of selenium are attributed to selenoproteins, proteins containing selenium in the form of the amino acid, selenocysteine (Sec). Sec insertion into proteins depends on Sec tRNA; thus, knocking out the Sec tRNA gene (*Trsp*) ablates selenoprotein expression. We generated mice with targeted removal of selenoproteins in keratin 14 (K14) expressing cells and their differentiated descendents. The knockout progeny had a runt phenotype, developed skin abnormalities and experienced premature death. Lack of selenoproteins in epidermal cells led to the development of hyperplastic epidermis and aberrant hair follicle morphogenesis, accompanied by progressive alopecia after birth. Further analyses revealed that selenoproteins are essential antioxidants in skin and unveiled their role in keratinocyte growth and viability. This study links severe selenoprotein deficiency to abnormalities in skin and hair and provides genetic evidence for the role of these proteins in keratinocyte function and cutaneous development.

## Introduction

Mammalian skin consists of epidermal and dermal layers separated by the basement membrane. The epidermis consists of multiple layers of stratified squamous epithelium composed primarily of keratinocytes. The basal layer cells of the epidermis interact with the basement membrane through hemidesmosomes and are capable of proliferation. Their progenies differentiate to generate the suprabasal spinous and granular layers and the stratum corneum [1]. The dermis is mesenchymally derived and is composed primarily of fibroblasts, dense dermal matrix, as well as other cell types; e.g. endothelial cells of blood vessels, fat cells, and macrophages. Hair follicle cells are of epidermal origin, which under the influence of specialized dermal cells develop in the dermis while maintaining their connection to the epidermis. Keratinocyte differentiation and interactions with the basement membrane and with neighboring cells within the epidermis play crucial roles in the structural integrity and development of skin.

Positioned at the interface between the body and the external environment, skin maintains body homeostasis and protects it from water-loss, injury, radiation and infections. It is constantly exposed to oxidative environmental stresses from UV rays, chemicals, air pollutants and microorganisms [2], [3]. These agents generate reactive oxygen species (ROS), which are involved in several skin disorders including skin cancer [4], [5]. The generation and neutralization of ROS is a continuous process in skin accomplished by non-enzymatic and enzymatic antioxidant substances, which protect the skin from the harmful effects of ROS [5]. Some well characterized antioxidant substances in human skin include beta-carotene, vitamin C, and vitamin E, and antioxidant enzymes glutathione peroxidases, copper-zinc superoxide dismutase, manganese superoxide dismutase and catalase [6], [7], [8], [5]. Some of these enzymes require trace elements for activity either as co-factors or as an integral component of proteins. Diets rich in these elements and compounds have a protective effect on skin, establishing the importance of dietary micronutrients in skin function [9], [10].

Selenium is a dietary micronutrient with chemopreventive and anti-cancer properties [11]. It is important for the proper function of many organs, including skin. Selenium protects against UVB-induced skin damage and cancers by 1) decreasing oxidative DNA damage; 2) preventing the production of immunosuppressive cytokines; and 3) enhancing the cellular and humoral immunity [9]. Studies have demonstrated that selenium deficiency adversely affects protective antioxidant enzymes in skin and increases tumor incidence in UVB exposed mice [12]. Topical application of carotenoids, vitamin E and selenium increases skin density and thickness and improves skin surface parameters such as scaling and roughness in humans [13]. Selenium-deficient rats display a slower growth rate and sparse hair growth in pups [14]. Selenium deficiency in an 18-month-old male led to dry skin, erythematous changes on cheeks, hips and thighs along with sparse, short, thin, light-colored hair [15]. Interestingly, the skin lesions disappeared upon dietary supplementation of selenium. Trace element supplementation using copper, zinc and selenium had a beneficial effect on wound healing along with a decrease in skin protein catabolism [16].

Many of the protective effects of selenium are thought to be mediated by selenoproteins that contain selenium in the form of the amino acid, selenocysteine [17]. Studies have identified 25 selenoprotein genes in humans and 24 in mice [18]. Incorporation of selenocysteine into proteins depends on its unique tRNA, Sec tRNA, the only known molecule of its kind in eukaryotes that governs expression of a single class of proteins, the selenoproteins [17]. As a result, knocking out the Sec tRNA gene (*Trsp*) ablates expression of all selenoproteins. *Trsp* knockout mice are embryonic lethal [19], [20]; hence, selenoprotein function must be studied by targeting the removal of *Trsp* in specific tissues. Though human skin cells express about 10–15 different selenoproteins [21], their role in skin is poorly understood. In order to elucidate their involvement in skin development and function, epidermal knockout of *Trsp* was targeted using *loxP-cre* technology wherein the *cre* recombinase is under the control of the keratin-14 (K14) promoter, which is active in basal cells of several stratified epithelia, including skin. This study reports that K14 promoter-driven loss of *Trsp* generates progeny with postnatal skin abnormalities, stunted growth and premature death. Lack of selenoproteins in epidermal cells of skin causes progressive alterations in epidermal differentiation after birth and aberrant hair follicle morphogenesis and hair formation, leading to progressive hair loss after birth. This study highlights the antioxidant role of selenoproteins in skin in addition to keratinocyte adhesion and growth in culture, reporting a crucial role of selenoproteins in cutaneous development and function. We also establish that dearth of selenoproteins bring about most of the abnormalities associated with selenium deficiency in skin.

## Results

### Generation of mice with restricted deletion of *Trsp* gene in keratin-14 (K14) expressing cells

To examine the role of selenoproteins in skin function and development, we generated mice with a keratinocyte-specific deletion of the *Trsp* gene, using *loxP-cre* technology. Floxed *Trsp* mice [20] in a C57BL/6 background were intercrossed with K14-*cre* recombinase mice in a FVB background. The resultant K14-*cre*; *Trsp^fl/+^* offspring were phenotypically normal and fertile. K14-*cre*; *Trsp^fl/+^* males were subsequently crossed to *Trsp^fl/fl^* females to prevent the maternal deleter phenotype due to expression of *cre* in oocytes of K14-*cre*–positive females [22]. This breeding scheme was used for maintenance of mice; hence the mice used in this study were in a mixed background of FVB and C57BL/6. A total of 586 pups were generated from crosses between K14-*cre*; *Trsp^fl/+^* and *Trsp^fl/fl^* and their resulting genotypes and phenotypes are shown in Table 1. Pups with deletion of the *Trsp* gene in keratinocytes (K14-*cre*; *Trsp^fl/fl^*) were designated as knockout, while littermates carrying heterozygous floxed *Trsp* gene and K14-*cre* (K14-*cre*; *Trsp^fl/+^*) served as controls. Knockout mice were born with the expected 25% Mendelian ratio (136 affected mice of 586 total; 23.2%; see Table 1).

**Table 1:** Offspring generated from mating K14-*cre*; *Trsp^fl/+^* males and *Trsp^fl/fl^* females.

Genotype	Number of pups (%)	Phenotype
*Trsp^fl/+^* *–*	146 (24.9)	Normal
*Trsp^fl/+^* *K14-cre*	194 (33.1)	Normal
*Trsp^fl/fl^* *–*	110 (18.8)	Normal
***Trsp^fl/fl^*** **** ***K14-cre***	**136 (23.2)**	**Skin abnormality, die in <2 wk**
Total586 (100)		

### Deletion of *Trsp* in K14 expressing cells results in stunted growth and neonatal lethality

**Figure 1:**
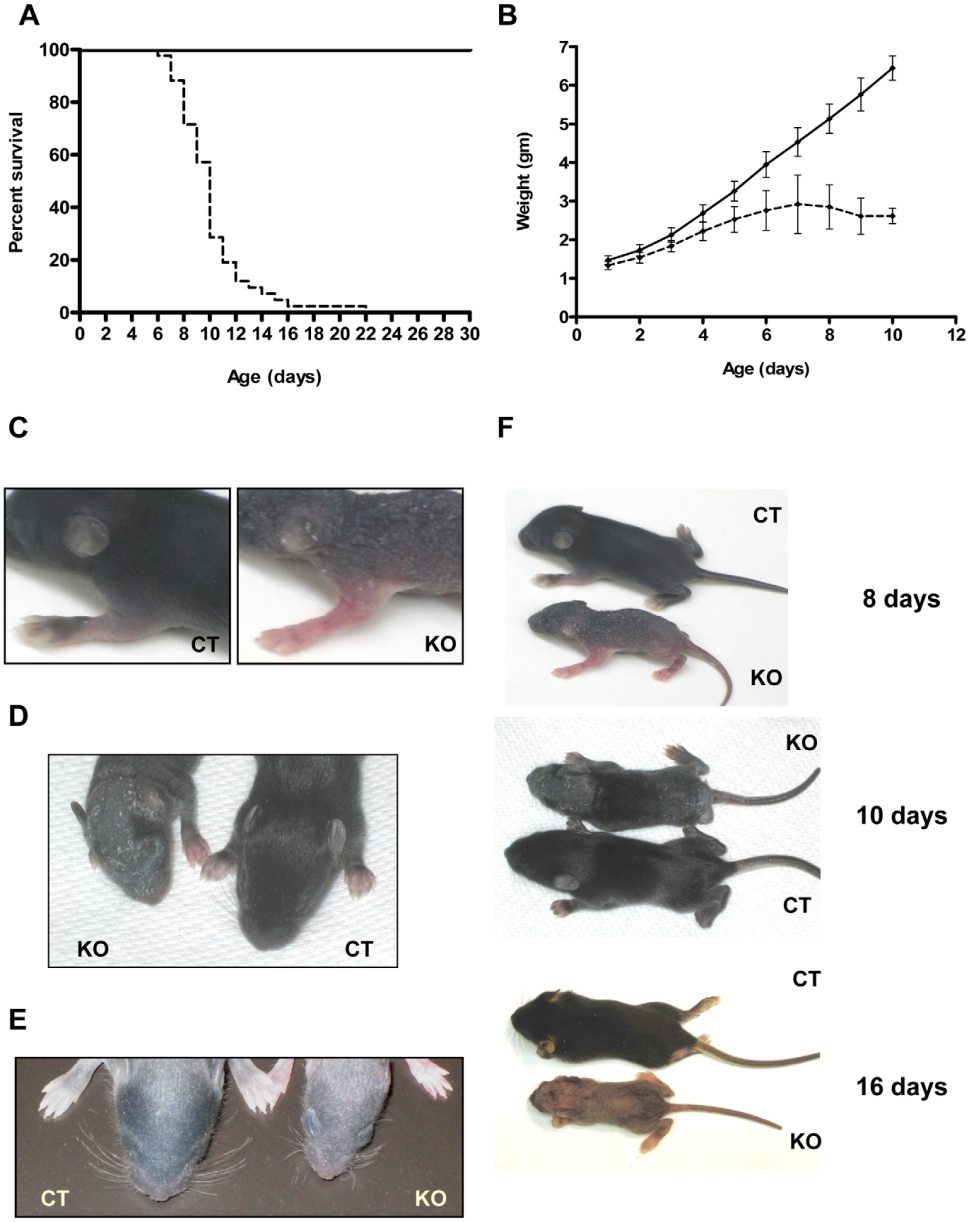
Neonatal mortality and runt phenotype in keratin-14 expressing epithelial cells encoding a *Trsp* deletion. (A) Kaplan-Meier survival plot of *Trsp* knockout (– – –, *n* = 42) and control mice (——, *n* = 51) (*p*<0.0001). (B) Growth curve of knockout (– – –) and control (——) littermates (*p*<0.01). (C–D) Phenotype of knockout (KO) mice showing fragile, flaky and wrinkled skin with sparse hair compared to control (CT) littermates. (E) Picture showing short and oddly angled vibrissae of whisker pads in knockout mouse compared to a control littermate at day 9. (F) Photograph showing that knockout mice are smaller and exhibit alopecia in comparison to control littermates of same age.

Knockout mice were indistinguishable from control littermates at birth, but exhibited a progressive runt phenotype and usually died within two weeks after birth, with their median survival being only 10 days (Figure 1A). The knockout pups failed to grow normally in spite of milk in their stomachs, observed following autopsy. However, milk-content in the stomach of knockout mice was less in comparison to control littermates. Monitoring the body weight of 35 pups from four litters (containing 12 knockouts) from day 1 until day 10 revealed that with age, knockout pups gained weight more slowly than control pups and began losing weight from day 7 (Figure 1B). In addition, the skin of these mice appeared flaky and wrinkled by day 6 (Figure 1C) and displayed defects in pelage hair growth in addition to short and misshapen vibrissae (Figure 1D and 1E). When control animals developed their hair coat, the knockout animals had sparse hair with partial alopecia, which increased with age, leading to almost total alopecia by day 16 (Figure 1F). The flakiness and fragility of skin was more severe near the neck and tail region of knockout mice, possibly due to mechanical stress which sometimes resulted in small wounds. It is worth noting that neonatal lethality and body weights varied considerably among knockout pups, even if they were within the same litter.

### *Cre*-mediated recombination deletes *Trsp* in a tissue specific manner

**Figure 2:**
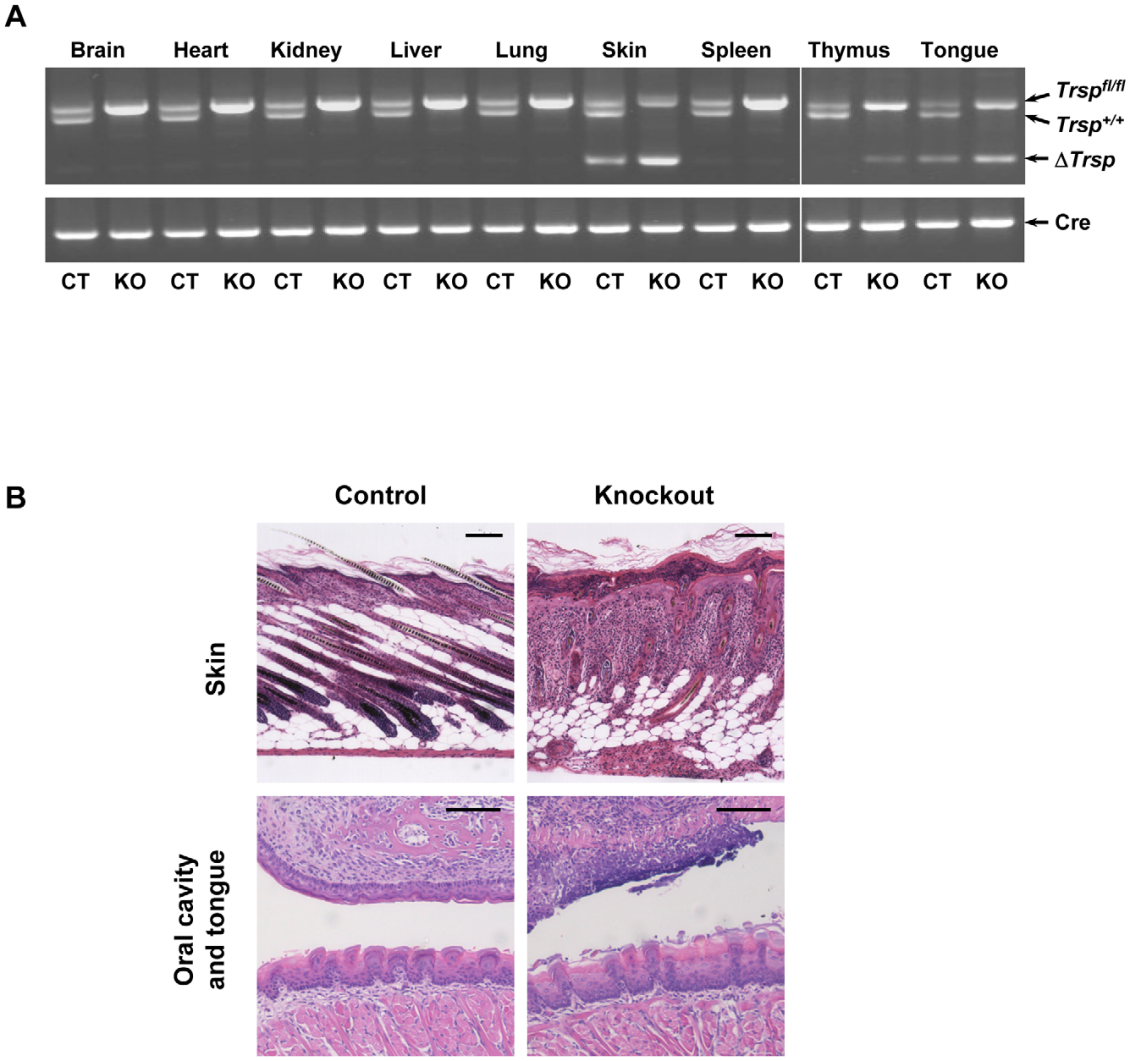
Tissue-specific detection of *Trsp* and histological examination of affected tissues. (A) Genotyping PCR of various tissues from knockout mice (KO) and control (CT) littermates is shown. *Trsp^fl/fl^* (upper band), *Trsp^+/+^* (center band) and Δ*Trsp* (lower band) products in the upper panel were 1100, 900 and 450 bp, respectively, and the amplified product from *cre* was 700 bp (lower panel). (B) H&E staining of skin, oral cavity and tongue from 8 day old knockout mice shows abnormalities in skin, oral cavity and tongue in comparison to control mice. Scale bars: 100 µm.

To determine the extent of recombination of *Trsp* in several tissues including skin, we extracted genomic DNA from various organs of knockout mice and their littermate controls. Genotyping knockout and age matched control mice showed that the Δ*Trsp* allele (450-bp product) was detected in skin and tongue of both genotypes, while Δ*Trsp* allele was observed in the thymus of knockout mice (Figure 2A). Partial recombination in control animals was expected due to the presence of K14-*cre*. The 450-bp, Δ*Trsp* product was not detected in any other tissue sample from control or knockout animals, establishing that *cre*-mediated recombination has taken place in the tissue-specific manner in mice with K14-*cre* (Figure 2A).

Histological examination of organs from knockout mice (Figure 2B) revealed that skin has reduced subcutaneous fat cells in addition to a thickened epidermis and aberrant hair follicles. There was a marked cellular infiltrate in the dermis. The oral mucosal epithelia were dysplastic with multifocal epithelial necrosis and the tongue epithelia were focally necrotic with hyperkeratosis. Though no gross abnormality was observed in the other examined organs, pathological examination of thymus of some knockout mice had moderate lymphocytolysis, which was speculated to be associated with stressful events resulting from severe effects in skin (data not shown).

### Selenoprotein mRNA profile in epidermis, dermis and cultured keratinocytes and comparative selenoprotein expression in freshly isolated keratinocytes from control and knockout mice

**Figure 3:**
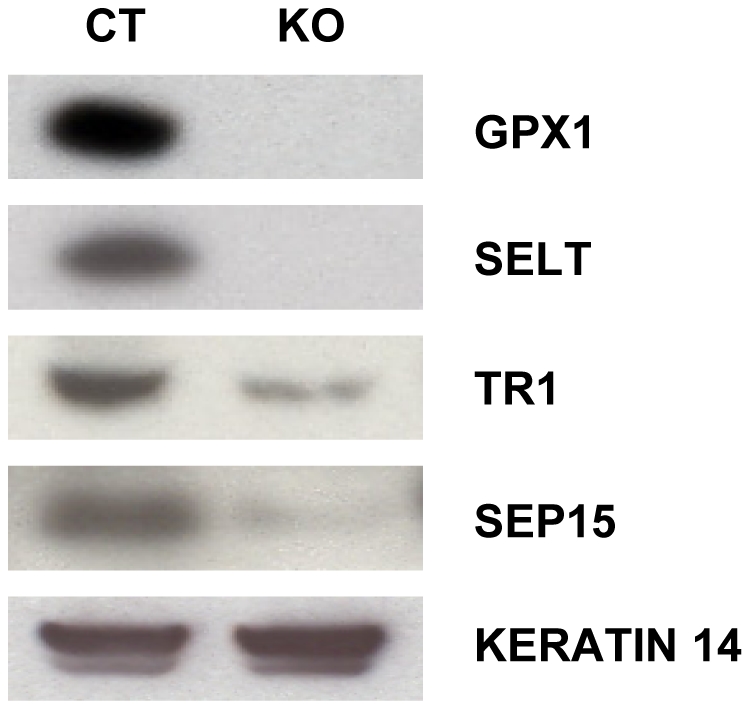
K14-mediated *Trsp* deletion ablates selenoproteins in epidermis of knockout mice. Loss of selenoproteins in freshly isolated keratinocytes from new born mice was detected by western blotting with antibodies against GPX1, SELT, TR1 and SEP15.

We examined the expression profiles of selenoprotein mRNAs in epidermal and dermal fractions of control mouse skin which demonstrated specific mRNA expression in the two layers of skin tissue (Figure S1). Glutathione peroxidases 1 and 4 (Gpx1 and Gpx4, respectively), selenoprotein 15 (Sep15), selenoprotein H, K, P, R, S, T (SelH, SelK, SelP, SelR, SelS and SelT, respectively) and thioredoxin reductase 1 (TR1) appeared to be the more abundantly expressed selenoprotein genes in mouse epidermis. However, Sep15 mRNA expression was higher in dermis, whereas the expression levels of other mRNAs was similar to that observed in epidermis, with the possible exception of SelP which appeared to be synthesized more in dermis (Figure S1). Since keratinocytes are the major constituent of the epidermis, we examined selenoprotein mRNA expression in cultured keratinocytes from control mice and the profile was very similar to that observed in epidermis. In order to determine the consequence of *Trsp* deletion, freshly isolated keratinocytes were used for immunodetection of several key selenoproteins. GPX1, SELT, TR1 and SEP15 were highly expressed in keratinocytes from control mice and virtually absent in the corresponding *Trsp* knockout cells (Figure 3). The faint bands observed in Δ*Trsp* keratinocytes were most certainly due to contaminating cells as further discussed below.

### Mice lacking selenoproteins in keratinocytes develop marked changes in epidermal differentiation and in hair follicle development and cycling

**Figure 4:**
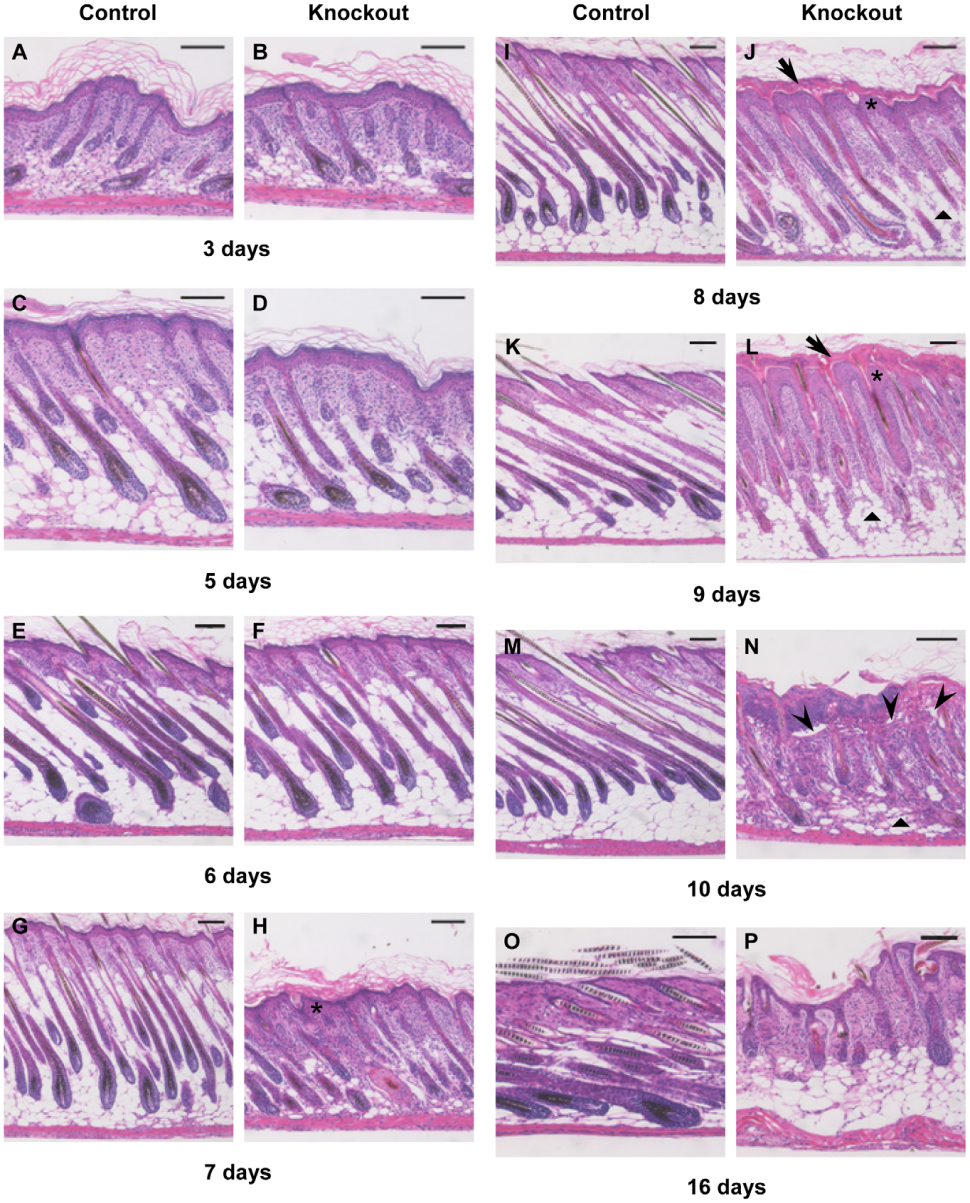
Progressive epithelial dysfunction and aberrant follicular morphogenesis in knockout mice. (A–P) Back skin sections from control (A, C, E, G, I, K, M, O) and knockout mice (B, D, F, H, J, L, N) were stained with H&E for histological examination. Thickened cornified layer (arrow), hyperplastic epidermis (asterisk) and detachment along dermal–epidermal junction (arrowhead) along with decrease in fat cells (triangle) is evident in knockout mice. Defective hair follicle morphogenesis in mutant skin is characterized by irregularly spaced malformed hair follicles. Scale bars: 100 µm.

Histological and immunohistochemical studies were performed to understand the underlying anomaly associated with abnormal skin and hair development in knockout mice. Histological examination of mouse back skin sections was carried out at different time points from day 3 through day 16 (Figures 4A–4P). The epidermis of knockout mice was morphologically normal at birth and similar to control mice until day 5 (compare Figures 4B and 4D to Figures 4A and 4C), The epidermis progressively became hyperplastic (asterisk) beginning at days 7 through 10 (Figures 4H, 4J, 4L and 4N), and by day 8, the knockout pups developed a thickened cornified-layer (arrow) compared to their control littermates (compare Figures 4J and 4L to Figures 4I to 4K). In knockout mice, the epidermis was often detached focally along the dermal-epidermal junction (arrowhead), gaining prominence by day 10 (Figure 4N) and detachment of the thickened cornified layer becoming obvious by day 16 (Figure 4P). The initiation of hair follicle growth and elongation appeared normal in knockout mice (day 3–5); however, by day 6 (Figure 4F), hair formation is reduced in knockout follicles (Figure 4F) compared to control skin (Figure 4E) and morphological changes in many hair bulbs resemble those described for early “dystrophic catagen” induced by high dose treatment of cyclophosphamide of C56BL/6 mice [23]. From day 7 on, hair follicles of mutant mice showed gross structural abnormalities often accompanied by a decrease in subcutaneous fat in the dermis (triangle). At day 9 (Figure 4L), hair follicles of control mice (in anagen phase) were uniformly spaced, aligned at a specific angle relative to the skin and grew deeply into subcutaneous fat. In comparison, follicles of mutant mice were irregularly spaced and showed gross abnormalities along with premature regression of follicles and a marked decrease in subcutaneous fat (triangle). At day 16, only follicular cysts were detectable in knockout mice (Figure 4P).

**Figure 5:**
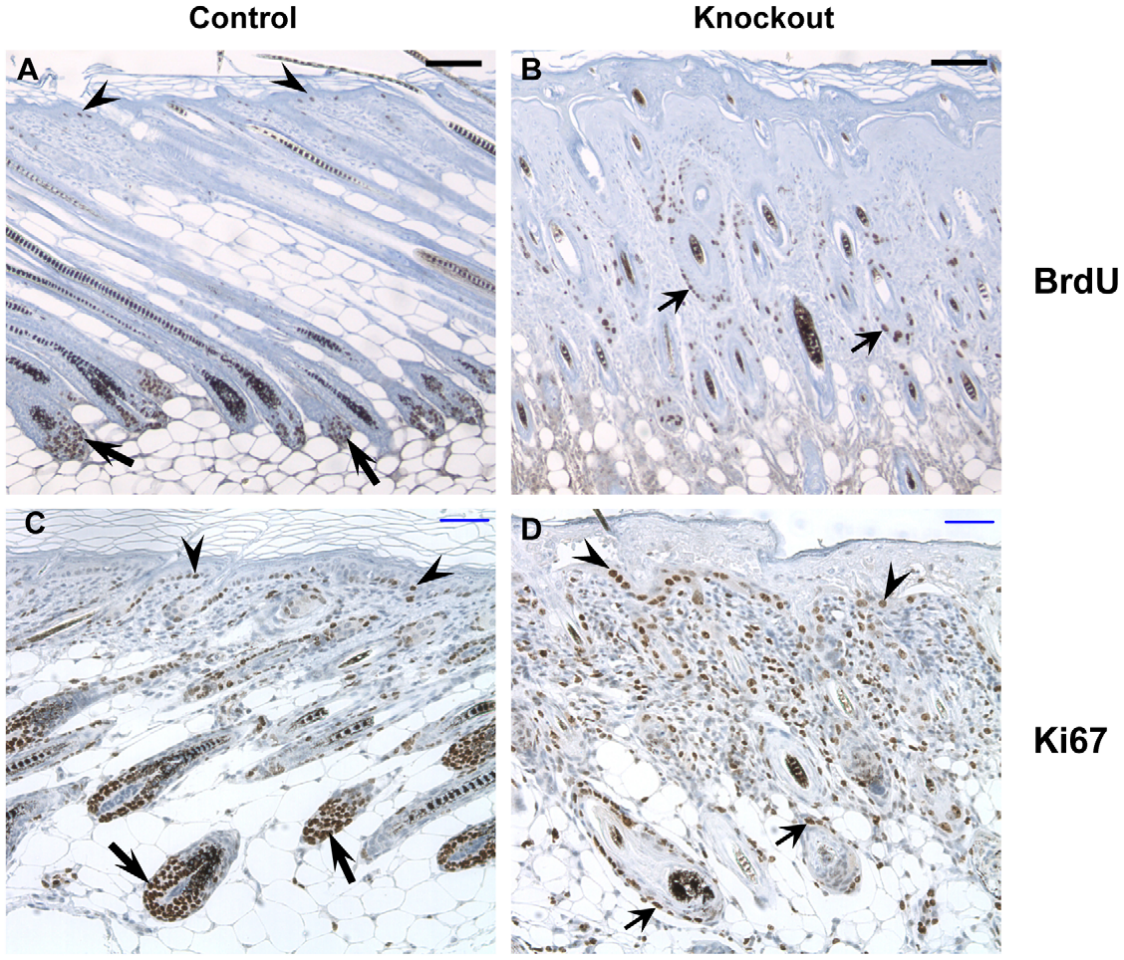
Mice lacking selenoproteins in keratin-14 expressing cells show reduced proliferation of hair matrix cells. (A–B) BrdU was administered intraperitoneally in 9 day old control and knockout mice and immunodetected for incorporation in back skin sections. (C–D) Ki67 staining for proliferating cells in skin sections of 10 day old control and knockout mice. Proliferating cells were noted in the basal layer of epidermis (arrowhead), hair follicle bulb (thin arrow) and outer root sheath of hair follicle (broad arrow). Scale bar: 100 µm.

Defects in epidermis and hair follicle morphogenesis in skin of *Trsp* knockout mice prompted us to examine the proliferation patterns in keratinocytes, indicated by expression of the proliferation markers, bromodeoxyuridine (BrdU) and Ki67 in skin of 9-day and 10-day old mice respectively (Figure 5). In back skin sections from control mice, proliferating cells were detected in the basal layer of epidermis (arrowhead) and in matrix cells of the hair follicle bulbs (thin arrow) (Figure 5A, 5C). In contrast, proliferation markers were detected in the outer root sheath (ORS) of hair follicles (broad arrows) of knockout mice, but functional matrix cells appeared to be absent in the abnormal follicles at that age (Figure 5B, 5D). The exact location of basal keratinocytes and hair follicle bulbs was difficult to discern in knockout mice due to malformed follicles and hyperthickened epidermis. Interestingly, Ki67 staining also revealed a hyperproliferative dermis in knockout mice.

### Lack of selenoproteins in epidermal cells alters the expression of differentiation marker proteins *in vivo*

**Figure 6:**
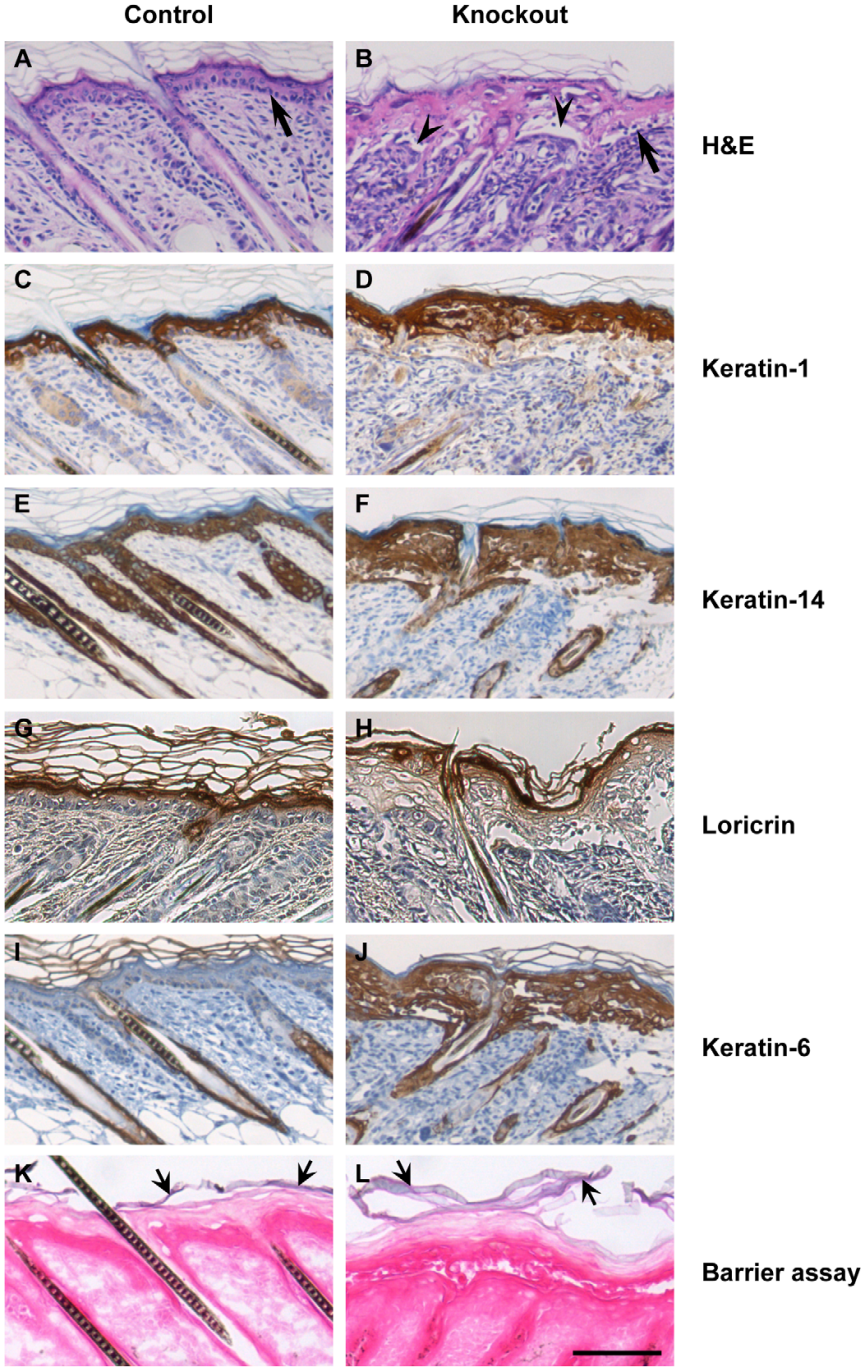
Abnormalities in keratinocyte morphology and differentiation. (A–B) H&E staining of back skin section of 10 day old control and knockout mice. Arrows indicate basal keratinocytes and arrowheads indicate blistering between epidermis and dermis. (C–J) Back skin sections of 10 day old control and knockout mice were stained for keratinocyte differentiation marker proteins. (C–D) keratin-1; (E–F) keratin-14; (G–H) loricrin; (I–J) keratin-6. (K–L) Barrier function of skin was tested using a hematoxylin dye penetration assay, showing hematoxylin staining only in stratum corneum (broad arrow). Scale bar: 100 µm.

Inability of knockout keratinocytes to synthesize selenoproteins lead to the development of a hyperthickened epidermis with frequent detachment along the dermal–epidermal junction (Figure 6B, arrowhead), clearly evident in the skin of 10 day old mice. The basal keratinocytes in the skin of control mice are arranged in a single layer of tightly packed cuboidal cells (Figure 6A, arrow), while in knockout mice, this layer was often less defined, or disrupted (Figure 6B, arrow). The differentiation status of keratinocytes in knockout epidermis was assessed by examining the expression of the stratified epithelial marker protein keratin-14 (K14), the suprabasal marker keratin-1 (K1) and the terminal differentiation marker loricrin. Keratin-1 was detected in the suprabasal layer of both the control and knockout skin (Figure 6C and 6D), while keratin-14 was uniformly expressed in the ORS of hair follicles and in the epidermis (Figure 6E and 6F). Expression of loricrin was confined to the cell layer just below the stratum corneum, both in control and knockout mice (Figure 6G and 6H). Interestingly, keratin-6 (K6), which is normally expressed in the ORS of hair follicles, but not in interfollicular epidermis (Figure 6I), was found throughout the epidermis of knockout mice (Figure 6J), highlighting a histological abnormality.

The wrinkled and flaky skin coupled to progressive loss in body weight of knockout mice prompted us to examine whether these mice underwent dehydration due to a defective barrier function in skin. The mice were subjected to a modified barrier dye penetration assay to determine skin permeability as described in Materials and Methods. Hematoxylin penetrated the superficial, but not the lower layers of the stratum corneum of both control and knockout mice (Figure 6K and 6L, broad arrows), suggesting an intact barrier function in skin.

In order to determine the progression of changes in differentiation patterns of keratinocytes as a function of age, we stained skin sections of 3, 5, 6, 7, 8, 9 and 16 day old control and knockout mice with antibodies to keratin-1, keratin-14 and keratin-6 (Figure S2 and Supplementary Information Figure legends). Keratin-1 expression was confined to the suprabasal layer in control mice of all ages, but extended to some hair follicles of knockout mice beyond 7 days of age (Figure S2A), while keratin-14 was detected in all layers of control and knockout epidermis as well as in the outer root sheath (ORS) of hair follicles (Figure S2B). In addition to the location in the ORS of hair follicles, keratin-6 was expressed in the suprabasal layer of 7, 8 and 9 day old skin from knockout mice, but confined to the abnormal hair follicles and cysts of 16 day old mice, with marginal staining in the epidermis which is no longer hyperplastic at that stage (Figure S2C).

### Absence of selenoproteins in keratinocytes alters their adhesion *in vitro* and leads to infiltration of inflammatory cells in skin *in vivo*

**Figure 7:**
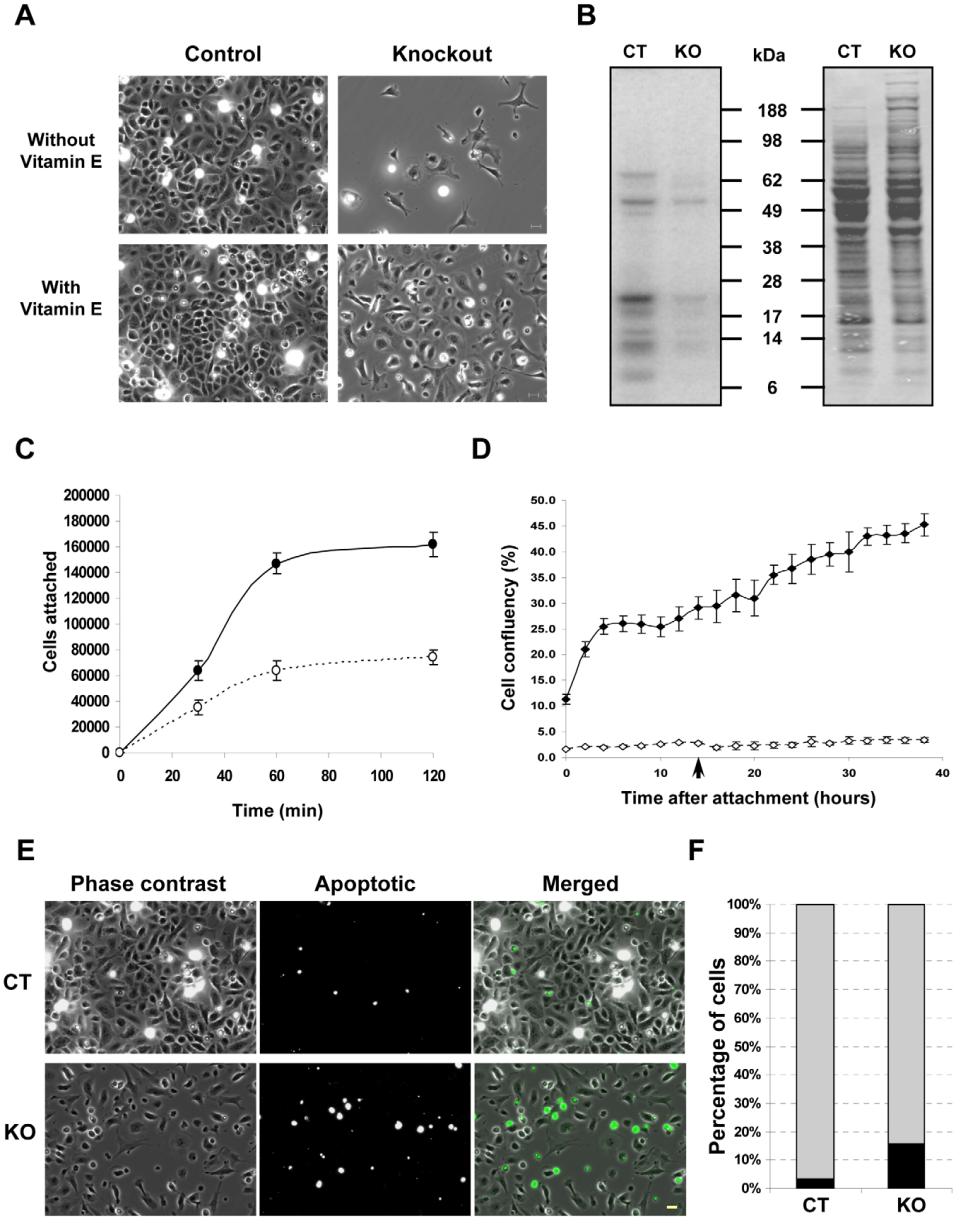
Selenoprotein deficiency alters adhesion and proliferation of keratinocytes in culture. (A) Attachment and proliferation of keratinocytes from knockout mice following 3 days of culture in ColI/FN coated plates is improved in vitamin E supplemented media. (B) Expression of selenoproteins in keratinocyte cultures from control and knockout mice. Left panel, incorporation of ^75^Se in proteins; right panel, Coomassie blue staining. (C) Adhesion assay for keratinocytes from control (—•—) and knockout mice (– –○– –) following 30, 60 and 120 min of attachment ± S.D for each time point. (D) Growth of keratinocytes from control (—⧫—) and knockout mice (– –⋄– –) following attachment for 2 h. The unattached cells were removed and media changed following attachment, with replenishment after 14 h of attachment (arrow). The curve represents the mean values ± S.D. for each time point. (E–F) TUNEL assay for determining cell death by apoptosis in keratinocyte cultures from control (CT) and knockout (KO) mice. The abundance of apoptotic cells (depicted in black) is plotted as percentage of the total population. Scale bar: 20 µm.

Primary keratinocytes isolated from control and knockout mice were cultured to examine the effect of selenoprotein ablation on keratinocyte properties in culture. While keratinocytes from control mice adhered and proliferated on tissue culture plates without any supplemented coating, those from knockout mice failed to attach in significant numbers (data not shown). Speculating that the adhesion of keratinocytes from knockout mice might be compromised, we used tissue culture plates coated with collagen IV, Matrigel, or a mixture of collagen I and fibronectin (ColI/FN) [24]. We observed that keratinocytes from knockout mice attached and grew best on ColI/FN coated plates (data not shown). Thus, ColI/FN coated plates were used for all subsequent experiments. Although keratinocytes isolated from knockout mice attached and grew on ColI/FN plates, their confluency after 3 days of growth following attachment was very low. Supplementation with vitamin E significantly improved their confluency (Figure 7A). Selenoprotein-deficient keratinocytes exhibited altered morphology with spindle shaped and spread out cells, which is reminiscent of fibroblasts, rather than the characteristic cobblestone-like morphology of epithelial cells (Figures 7A and S3A). The vitamin E supplemented keratinocytes isolated from control and knockout mice were labeled with ^75^Se to visualize the expression of selenoproteins. Though most of the selenoproteins were virtually absent in the knockout preparation, some selenoproteins were still visible in the preparation (Figure 7B).

As the primary keratinocyte preparation contains both keratinocytes and melanocytes and an earlier study on human skin cells has shown a preferential expression of selenoproteins in melanocytes [21], we investigated the possibility that the visible selenoprotein bands were contributed by non-keratinocytic cells, and most likely mainly melanocytes. Cells from control and knockout mice grown on ibidi™ slides, immunostained for keratin-14 and counterstained for nuclei (Figure S3A) revealed that the proportion of non-keratinocytic cells in the knockout preparation was 3-fold higher than control cells (Figure S3B); hence, the latter cell types were likely responsible for the selenoproteins seen in cultures isolated from knockout mouse skin (Figure 7B).

Altered morphology and failure of the cells to grow on untreated cell culture plates suggest that adhesion may have been impaired in these cells (Figure S3A). To investigate attachment and subsequent proliferation of keratinocytes, an adhesion assay [25] was carried out, where cells were allowed to adhere for various time points following plating. Our observations indicated that 53% of the cells from control mice adhered after 120 min, whereas only 25% cells from knockout mice adhered to ColI/FN coated plates (Figure 7C). Following attachment, media were changed and the cells were allowed to proliferate. Proliferation was monitored by time-lapse imaging of culture area occupied by attached cells; with the media being changed again after 14 h (arrow). Keratinocytes from control mice reached a confluency of 45% after 38 h, compared to 4% for those from the knockout mice (Figure 7D and S4), indicating marked differences in spreading, morphology, and growth of selenoprotein-deficient keratinocytes. Cultured keratinocytes from knockout mice had many rounded cells, which prompted us to examine this population for apoptotic cells. The number of apoptotic cells determined by *in situ* TUNEL assay was also substantially increased in selenoprotein deficient keratinocytes (Figure 7E and 7F).

**Figure 8:**
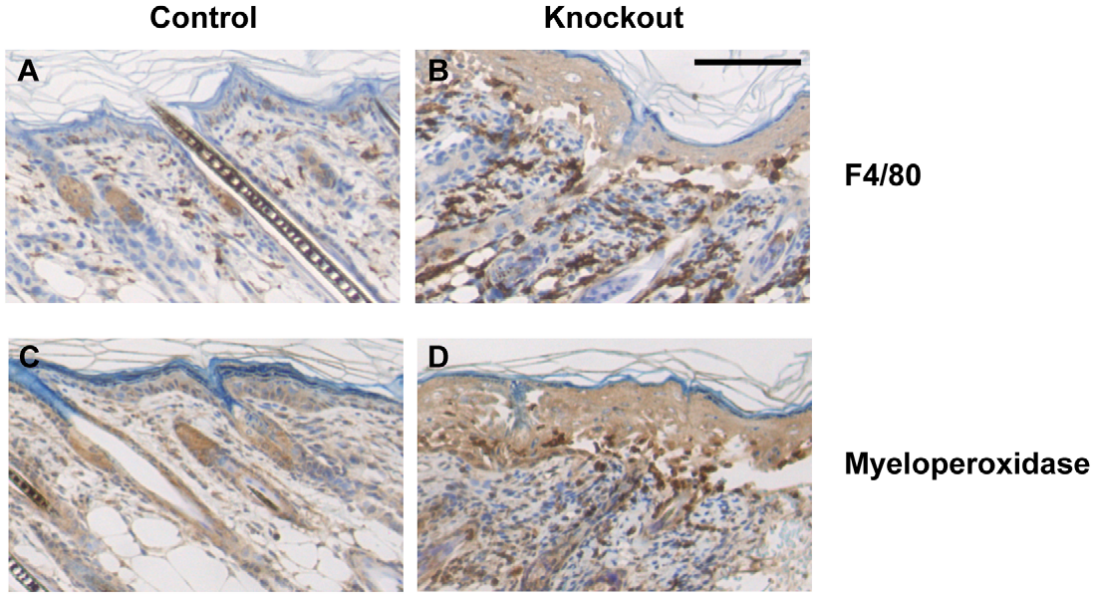
Loss of selenoproteins induces infiltration of inflammatory molecules and alters cell-adhesion in knockout mice skin. (A–B) Immunostaining for infiltrating macrophages with macrophage/monocyte specific antigen F4/80 in 10 day old mouse back skin section. (C–D) Detection of granulocytes with myeloperoxidase staining in back skin section of 10 day old mice. Scale bar: 100 µm.

Defective keratinocyte adhesion and malformed hair follicles can trigger a wound healing response in skin, leading to infiltration of inflammatory cells. To test this possibility, we analyzed skin sections for the presence of granulocytes and macrophages. Macrophage infiltrates were significantly increased in knockout skin samples around some deformed hair follicles and at areas of dermal-epidermal separation (Figure 8B) in comparison to control mice (Figure 8A). This infiltration was enhanced by day 7 and continued through day 10, but the macrophage population was reduced by day 16 (Figure S5). Granulocytes, which are rarely detected in control skin (Figure 8C), were also abundant in knockout skin mostly at the dermal-epidermal junction and around malformed hair follicles (Figure 8D and S6). The presence of granulocytes was detected as early as day 8, while their abundance was more prominent on days 9 and 10 (Figures S6 and 8D). At 16 days, granulocytes were primarily detected near deformed follicles (Figure S6). The accumulation of macrophages and granulocytes around some deformed follicles and at the sites of dermal-epidermal separation suggested that these inflammatory cells might play a role in removing the deformed follicles and detached epidermis that arose due to the lack of selenoproteins in keratinocytes. To examine if the separation of epidermis from the basement membrane, and failure of keratinocytes to adhere to culture plates was related to the expression of integrins, we studied the expression of beta 1 integrin in tissue sections (Figure S7A). The staining was intense in knockout skin sections, with integrin beta 1 being detected in the suprabasal layer of knockout skin (Figure S7A), while elevated levels of integrin beta 1 in lysates from freshly isolated knockout keratinocytes corroborate our observation (Figure S7B).

### Mice lacking selenoproteins in keratinocytes permits ROS accumulation and lipid peroxidation in these cells

**Figure 9:**
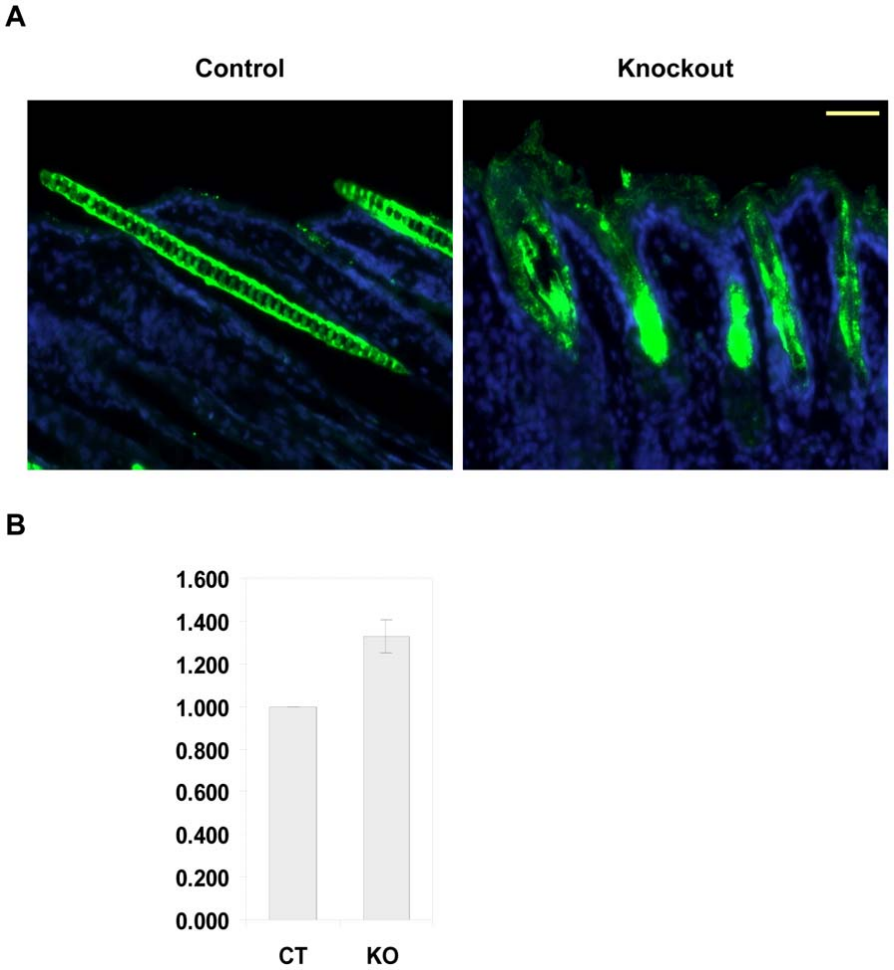
Selenoprotein ablation generates ROS and induces lipid peroxidation in knockout mice skin. (A) Immunofluorescent detection of DCFH-DA to measure ROS in 14 day old mouse back skin section. DCFH-DA is stained green and nucleus is stained blue with DAPI. (B) Measuring lipid peroxidation through ELISA assay for 4-hydroxynonenal (4-HNE), a byproduct of lipid peroxidation in protein lysates from freshly isolated keratinocytes from control (CT) and knockout (KO) mice. The data represents the mean values ± S.D. for three independent experiments carried out in duplicate. Scale bar: 50 µm.

Most selenoproteins are involved in redox regulation, hence we wanted to examine if their loss generates ROS in skin. Skin sections were treated with DCFH-DA and examined under a fluorescence microscope. Intense staining was observed not only in the deformed follicles but also in the epidermis of knockout mice, whereas in the skin of littermate control mice specific ROS staining was absent (Figure 9A). The staining of hair in control skin is non-specific and is due to autofluorescence of the mature hair shaft. ROS generation can also be accompanied by lipid peroxidation of cell membranes and organelles, resulting in cellular damage. 4-hydroxynonenal (4-HNE), a toxic aldehyde, is the byproduct of lipid peroxidation and a sensitive marker of oxidative damage and lipid peroxidation. Detection of 4-HNE in lysates from freshly isolated keratinocytes through ELISA showed a 1.3 fold increase in knockout mice lysates (Figure 9B), substantiating that the lack of selenoproteins in keratinocytes allows excess ROS accumulation and lipid peroxidation.

## Discussion

To elucidate the role of selenoproteins in skin function, we targeted the removal of *Trsp* gene in mouse cells that express K14 and examined the phenotypic consequences of this deletion. The expression of *cre*-recombinase under the control of mammalian K5 or K14 promoter have provided a useful molecular tool in analyzing genes in the epidermis of mice [26], [22], [27], [28]. However, the *loxP-cre* mediated recombination of genes in mice containing these promoters is female germ-line mediated, which restricts the breeding strategy [22], [28]. The knockout mice developed scaly thick skin within 6 days after birth and manifested a progressive runt phenotype and premature death. The lethality and body weights varied considerably even among knockout pups from the same litter, which could be attributed to a possible difference in the efficiency of *cre*-mediated *Trsp* deletion or nutritional differences. Though the knockout pups appeared to be identical to control littermates at birth, skin and hair defects became apparent within 6 days after birth. Malformed hair follicles, hyperplastic epidermis, and dysplastic mucosal and tongue epithelia were clearly evident in 8 day old mice. The weight loss in the knockout mice started from day 7, while pups were still nursed by their mothers. Abnormalities in mucosal and tongue epithelia may hamper their suckling and these mice may not have an adequate intake of milk. Though this cannot be the sole cause of premature death, it is most certainly a contributing factor. We did not find any gross histopathological abnormalities in other organs. K14-*cre* mediated deletion of *Trsp* blocked the expression of Sec tRNA in keratinocytes, in turn preventing the synthesis of selenoproteins in this cell type. It should also be noted that selenoproteins are not only absent in K14 promoter active cells, but in all cells derived from K14 promoter active progenitors, which include differentiated keratinocytes of epidermis and hair follicles. An examination of some key selenoproteins by western blot revealed their virtual absence in keratinocytes from knockout mice.

As pointed out before, the loss of selenoproteins was not restricted to K14 promoter active cells, but to all descendents of such cells, including the suprabasal cells of epidermis and epithelial cells of hair follicles. Although hair follicle initiation and elongation appeared normal in mice lacking selenoproteins, a histological time course analysis revealed impaired hair shaft formation, changed hair follicle appearance, and early abnormal regression of follicles with a concomitant decrease in subcutaneous fat. Hair follicles are composed of multiple concentric layers of keratinocytes around the developing hair shaft arising from the proliferating matrix cells in hair follicle bulb. The most marked early morphological changes occur in the matrix cell region of follicles of knockout mice, suggesting oxidative damage leading to cessation of cell proliferation and premature onset of catagen. It is noteworthy that the proliferation markers Ki67 or BrdU were detected in the ORS of hair follicles, but strikingly absent in the area of the follicles where matrix cells are expected to be located at 9 days after birth. The ROS accumulation and lipid oxidation in knockout follicles detected in skin sections of 14 day old mice is likely to have started in younger mice. Damaged hair follicles can trigger inflammatory cells and we observed infiltrating macrophages in knockout skin from 7 day old mice and granulocytes in knockout skin from 8 day old mice. These cells were most abundant around grossly deformed hair follicles and may assist in the removal of damaged hair follicles by phagocytosis. The triggering event to initiate oxidative damage is so far unknown. Nevertheless, our observations emphasize the importance of selenoproteins in hair follicle morphogenesis in postnatal mouse skin.

Histological examination of the epidermal layer from knockout mice reveals the epidermis to be morphologically normal at birth, but gradually exhibiting a thickened cornified layer, along with progressive hyperplasia. Micro-blisters were observed in the epidermis by focal separation of cell layers within the epidermis as well as at the basement membrane, leading to detachment at the epidermal-dermal junction. This detachment in mice lacking selenoproteins may contribute to the fragility and flakiness of skin. The typically cuboidal morphology of basal keratinocytes, which express K14, is obscure in knockout mice. Ablation of selenoproteins in keratinocytes creates a wound-like condition in skin, marked by epidermal as well as stromal hyperplasia. The study of keratinocyte differentiation markers as a function of age shows that K1 expression extended to some hair follicles of knockout mice, while K6 was aberrantly expressed in the epidermis. The aberrant expression of K6 in epidermis is suggestive of chronic hyperproliferation or abnormal differentiation. K6, the ‘stress inducible’ keratin, is upregulated in wound healing [29], psoriasis [30], and is strongly expressed in squamous cell carcinomas [31]. A recent study reported that prolonged expression of K6, through UVB-induction leads to wrinkle formation [32]. ROS accumulation in hair follicle and epidermis is observed in knockout mice, indicating generation of stress which may induce the expression of K6, resulting in the observed epidermal phenotype. The elevated expression of K6 in knockout mouse epidermis could be a contributing factor to wrinkle formation and flakiness in skin.

Primary keratinocytes isolated from knockout mice and subsequently cultured had altered morphology, severe adhesion and growth defects and underwent apoptosis. Under standard culture conditions for mouse keratinocytes, the adhesion was severely compromised and the keratinocytes required plating on ColI/FN-coated culture surfaces for attachment and growth. Integrins are receptors for extracellular proteins, responsible for anchoring the epidermis to basement membrane. In skin, they also play important roles in wound repair, inflammation, keratinocyte differentiation and proliferation and their aberrant localization and expression is a crucial factor in numerous diseases and squamous cell carcinoma [33]. Suprabasal expression of integrin beta 1 in epidermis has been attributed to hyperproliferation of keratinocyte, alteration of differentiation, inflammatory response and pathogenesis of psoriasis [33]. The anomalous expression of integrin beta 1 and keratinocyte differentiation marker K6 suggests that loss of selenoproteins in skin contributes to psoriasis like conditions in the knockout progeny. In a recent study, we reported that the loss of selenoprotein T, a protein with role in redox regulation has been associated with impaired cell adhesion [34]. Western blot data shows a virtual absence of SelT in knockout keratinocyte lysates and this loss may play a role in adhesion defects, yet contribution from other selenoproteins cannot be ruled out. In addition, we have previously found that the loss of selenoproteins in macrophages led to both an increase in reactive oxygen species (ROS) and an aberrant expression of ECM-related genes [35], though the role of selenoproteins in adhesion warrants an elaborate investigation. Although the attachment and growth was improved by ColI/FN treatment of the culture surfaces, the expansion of keratinocytes from knockout mice was markedly reduced in comparison to those from control mice. However, expansion was improved by continued supplementation with vitamin E, an essential antioxidant present as α-tocopherol in murine and human skin [36], [37]. Vitamin E is a well characterized antioxidant in skin along with selenoenzymes such as glutathione peroxidases and studies have shown that topical application of vitamin E and selenium improves skin surface parameters [13]. We also observed that the percentage of non-keratinocytic cells (mainly melanocytes) was higher in preparations of keratinocytes from knockout mice, which could potentially generate oxidative stress, influencing the growth of keratinocytes. However, the improvement in growth by vitamin E supplementation in keratinocyte cultures from newborn mice emphasized the need for antioxidants in keratinocyte growth. Most selenoproteins that have been characterized thus far are antioxidants [18] and their absence most certainly makes keratinocytes prone to oxidative damage that in turn may influence their morphology, attachment and growth. Our hypothesis is supported by enhanced accumulation of ROS in skin sections of knockout mice. This emphasizes the critical antioxidant role of selenoproteins in epidermal function and keratinocyte growth.

The premature death of mice lacking selenoproteins in K14-expressing cells and their descendents is not clearly understood, but could be a cumulative effect of numerous abnormalities in these mice. Wrinkle formation and fragility are markers of photo aging, changing the integrity of skin, which in combination with alopecia and lack of subcutaneous fat can result in a hypothermic condition. In addition, the dysplastic mucosal and tongue epithelia could inhibit their suckling, in turn hampering adequate food intake, leading to weight loss in knockout animals. Though a definite cause cannot be singled out, in all probability, the reduced food uptake and grossly disturbed skin along with some hitherto unidentified internal defects could contribute to the reduced lifespan of mice lacking selenoproteins in K14-expressing cells.

In conclusion, we have shown that loss of selenoproteins in K14-expressing cells altered normal morphogenesis in skin and its appendages, including hair follicles within several days after birth, leading to premature death. Earlier studies have linked some abnormalities of skin and hair to selenium deficiency [14], [15] and have shown the protective role of selenium as antioxidant [12] and in wound healing [16]. In the present study we attribute selenoproteins to the protective roles of selenium in skin and establish that deficiencies in selenoproteins beget most abnormalities associated with selenium deficiency in skin. The current study unveils the importance of selenoproteins in keratinocyte growth and viability, providing genetic evidence for the role of selenoproteins in cutaneous function and development.

## Materials and Methods

### Ethics Statement

All mouse experiments were approved by the Animal Ethics Committee at the National Institutes of Health, under the approved protocol number BRL-005. Mice were handled and humanely sacrificed in accordance with the National Institutes of Health Institutional Guidelines under the expert direction of Dr. John Dennis (NCI, NIH, Bethesda, MD, USA).

### Generation and maintenance of KO mice

The mice analyzed in this study were generated by crossing *Trsp^fl/fl^* mice [20] with K14-*cre* transgenic mice [26]. The resulting *Trsp^fl/+^*; K14-*cre* male mice were then crossed with *Trsp^fl/fl^* females to generate *Trsp^fl/fl^* and *Trsp^fl/+^* offspring either with or without K14-*cre*. The same breeding pattern was followed thereafter for maintenance of offspring. The breeders were maintained on a selenium adequate diet [20].

### Genotyping of mice and analysis for tissue-specific recombination

Genomic DNA from tips of mouse tails was isolated and genotyped using the REDExtract-N-Amp™ Tissue PCR Kit (Sigma-Aldrich), according to manufacturer's instructions. PCR amplification with the combination of CKNO2 and 8RP primers (Table S1) yielded 900-bp and 1100-bp fragments for the wild type (*Trsp^+/+^*) and floxed alleles (*Trsp^fl/fl^*), respectively, and a 450-bp fragment for the Δ*Trsp* fragment. The Cre-Forward and Cre-Reverse primers were used for detection of the *cre* transgene in mice, amplifying a 700-bp product.

DNA samples extracted from various tissues collected at the time of autopsy were examined for tissue-specific recombination of the conditional allele by genotyping PCR as described above.

### Separation of dermal–epidermal sheets and isolation of RNA

Separation of dermal and epidermal sheets is based on a previously described procedure [38]. Briefly, back skin samples from 6–7 day old pups were floated on a 3.8% ammonium thiocyanate solution in PBS, pH 7.4, at room temperature for 30 min. Following incubation, the epidermis was separated from underlying dermis with watch-maker's forceps. Both the epidermal and dermal fractions were washed with PBS, ground to powder in liquid nitrogen and total RNA extracted using TRIZOL (Invitrogen), according to the manufacturer's protocol.

### Q-PCR (quantitative real-time PCR)

Two-step Q-PCR was performed to validate the relative expression of genes as described [39] using primer sequences outlined in Table S2. For each sample, 1 µg of total RNA was reverse transcribed using iScript™ cDNA Synthesis Kit, according to the manufacturer's protocol and used for qPCR in combination with respective primers and SYBR green supermix. Reactions were carried out in triplicate and primer specificity was confirmed by melting curve analysis. RNA levels were normalized to *Gapdh,* and the expression levels were compared with those of control mice.

### Protein isolation and western blotting

Protein was extracted from freshly isolated keratinocytes from newborn mice as described [40]. Following extraction, proteins were quantitated by BCA protein assay and resolved by SDS-PAGE. Resolved proteins were electro-transferred to polyvinylidene difluoride membranes (Invitrogen), blocked with Tris-buffered saline (TBS) containing 5% non-fat dried milk and 0.02% Tween 20 for 1 h at room temperature and incubated overnight at 4°C with the indicated antibodies. Specific binding was detected using corresponding horseradish-peroxidase-linked secondary antibodies followed by an enhanced chemiluminescence method (Thermo Fisher Scientific Inc.).

### Histological and immunochemical analysis

Knockout mice and age matching control littermates were sacrificed at various ages by CO_2_ inhalation. Skin was surgically excised and pieces of back skin were snap-frozen in liquid nitrogen and stored at −80°C until molecular analysis. For histological examination, skin pieces from identical regions of back skin were either immersed in OCT compound, rapidly frozen in liquid nitrogen and sections cut with cryostat or fixed in 70% ethanol or neutral buffered formalin (NBF) for paraffin-embedded sections. Mice were dissected for gross examination and various tissues collected for future molecular analyses and then the whole body was fixed in NBF. Samples were submitted for processing and histopathological examinations were carried out by staining tissue sections with hematoxylin-eosin (HE). The paraffin-embedded tissue sections were used for immunohistochemistry by deparaffinizing in xylene, followed by alcohol rehydration. After quenching endogenous peroxidases with Peroxidase block (Dako), slides were rinsed in PBS, and when required, an antigen retrieval step was carried out for 10 min in preheated citrate buffer (pH 6.0). Slides were subsequently incubated with primary antibodies at 4°C overnight. Primary antibodies against keratin-1 (Covance, PRB-165P), keratin-6 (Covance, PRB-169P), keratin-14 (Covance, PRB-155P; The Binding Site, PH503), loricrin (Covance, PRB-145P), Ki67 (Vector Laboratories, VP-K451), bromodeoxyuridine (Invitrogen, A21301MP), integrin beta 1 (BD Transduction Laboratories, 610467), F4/80 (CALTAG, MF48000) and myeloperoxidase (DAKO, A0398) were applied, followed by incubation with biotin-conjugated appropriate secondary antibody. The Vectastain Elite ABC kit and DAB (Vector Laboratories) were used for detection, following the manufacturer's instructions and the slides were counterstained with Mayer's hematoxylin.

### Measurement of reactive oxygen species (ROS) production and lipid peroxidation

Reactive oxygen species (ROS) production in skin sections was measured using the fluorescent dye, 2′,7′-dichlorofluorescein diacetate (DCFH-DA). Frozen sections from control and knockout mice were incubated with DCFH (10 µmol/L) and DAPI (nuclear stain) in PBS for 30 min at 37°C protected from light. Sections were washed once with PBS and fixed with 4% paraformaldehyde and photographed under Zeiss fluorescence microscope using a digital camera. A semi quantitative estimation of lipid peroxidation in lysates from freshly isolated keratinocytes was carried out using the Oxiselect™ HNE-His Adduct ELISA kit according to manufacturer's recommendations (Cell Biolabs Inc.). The experiments were carried out with three different sample sets in duplicate and data was plotted relative to values obtained for lysates from control sample.

### *In vivo* bromodeoxyuridine assay

To analyze the proliferative rate of keratinocytes *in vivo*, mice were injected intraperitoneally with approximately 1 ml/100 g body weight of bromodeoxyuridine (BrdU) labeling reagent (Zymed Laboratories). The animals were sacrificed 2 h post injection and tissue samples were collected and fixed either in 70% ethanol or 10% NBF. To exclude regional differences in proliferative activity, skin samples derived from identical areas of the back were excised.

### Skin permeability assay

A modified barrier dye penetration assay [41] was carried out to determine the barrier function of skin. Eight-day old mutant and control mice were sacrificed with carbon dioxide, fixed for 5 min with methanol followed by incubation for 4 h in 0.5% hematoxylin. After brief washing with water, skin pieces were dissected and snap frozen with OCT and cryosections were counterstained with eosin.

### Primary keratinocyte culture and analysis

Primary keratinocytes were isolated and cultured from newborn mice as described [24] with some modifications as follows: all media, both for preparation and culture of primary keratinocytes from control and knockout mice, were supplemented with 100 nM sodium selenite and 100 µM or 200 µM α-tocopherol (vitamin E). Keratinocytes were plated on Collagen I/Fibronectin (ColI/FN) coated culture surfaces [24]. Initial plating of primary keratinocytes was done in HiCa (1.3 mM Ca^2+^) medium for 2 h and following this attachment period, medium containing unattached cells was aspirated, attached cells were rinsed with LoCa (0.05 mM Ca^2+^) medium. From that point on cells were maintained in the LoCa medium and medium was changed daily. Plating 0.5 Mouse Equivalents (ME) of knockout keratinocytes per well in a 12-well plate yielded an approximately 70% confluent monolayer on day 3, while a similar cell density was achieved by plating 0.2 ME of control keratinocytes. Cultures were generally analyzed about 2 to 3 days after plating.

For immunocytochemistry and TUNEL assay, primary keratinocytes isolated from control and knockout mice were cultured on ColI/FN coated 8-well ibiTreat tissue culture slides (Ibidi). For immunodetection of keratin-14, cultured cells were fixed with 2% paraformaldehyde solution, permeabilized with 0.1% Triton-X-100 in PBS and blocked with 3% BSA in PBS. Slides were then incubated with rabbit anti-keratin-14 antibodies (Covance, 1∶10000) overnight at 4°C. Rhodamine-labeled anti-rabbit secondary antibody was used to probe the slides, and cells expressing keratin-14 were detected using a fluorescence microscope. The nucleus was counterstained with DAPI or propidium iodide (PI). Fluorescent images were taken at individual channels and merged with pseudo-colors for individual fluorophores. TUNEL staining for the detection of apoptotic cells was carried out on cultured cells according to the manufacturer's instructions (Roche). Total cell number was counted to calculate the percentage of TUNEL positive cells in at least 4 randomly selected areas of the slide.

To label selenoproteins with ^75^Se and visualize the labeling, primary keratinocytes isolated from control and knockout mice were seeded onto a 6-well plate at densities of 0.2 and 0.5 ME cells/well, respectively, and cultured for 3 days. The cells were washed once with PBS and labeled for 12 h with 50 µCi/ml of [^75^Se] in LoCa media at pH 7.2. Following incubation, labeled cells were harvested, washed with PBS and protein lysate prepared as described earlier. Following estimation of protein concentration, 40 µg of protein were electrophoresed on SDS-PAGE, stained with Coomassie Blue, dried, and exposed to a PhosphorImager as described [20].

### Cell adhesion assay and time lapse imaging of keratinocytes

For cell adhesion assay [25] and time lapse imaging of attached keratinocyte, equal number of keratinocytes from knockout and control mice were plated per well of ColI/FN coated 12-well tissue culture plates. Freshly isolated mouse keratinocytes were seeded in quadruplicate wells for each attachment time at a density of 3×10^5^ cells (approximately 0.04 ME) per well and incubated at 37°C for 30, 60 or 120 min, following which, the plates were gently swirled 2–3 times and the media containing unattached keratinocytes were collected. The number of unattached cells was determined using a Coulter counter (Beckman Coulter).

Following the 2 h attachment, unattached cells were removed, media changed as described earlier, and plates were transferred to an IncuCyte (Essen) incubator monitor, for automatic imaging at 2 h intervals. Cell confluency was measured by recording the cell numbers at nine selected points in each well, preset by the instrument, at different time points for 38 h.

## Supporting Information

Figure S1 Selective expression of selenoproteins in skin and in cultured keratinocytes. Expression profile of selenoprotein (SP) mRNAs in epidermis and dermis from 6–7 day old mice and primary keratinocytes (cultured for 3 days) was determined by qPCR. The expression level of each selenoprotein was plotted relative to Gapdh. Bars represent the mean values ± S.D for 3 independent experiments.
(0.23 MB TIF)

Figure S2 Time course analysis of keratinocyte differentiation markers. Histochemical detection of keratinocyte differentiation markers in back skin sections of 3, 5, 6, 7, 8, 9 and 16 days old mice. (A) Keratin-1 is detected in the suprabasal layer of control and knockout mice and some hair follicles of knockout mice beyond 7 days of age. (B) Keratin-14 is uniformly detected in the ORS of hair follicles and epidermis of the examined control and knockout mouse. (C) Keratin-6 is expressed in suprabasal layer of epidermis of 7, 8 and 9 day old skin sections from knockout mice in addition to its localization in ORS of hair follicles. Scale bar: 100 µm.
(7.02 MB TIF)

Figure S3 Determination of non-keratinocytic cells in primary keratinocyte preparation. (A) Immunofluorescence staining for keratin-14 in primary keratinocyte preparation from epidermal cell fraction of control and knockout mice, cultured for 3 days. Size bar: 20 µm. (B) The abundance of non-keratinocytic cells (depicted in black) in primary epidermal cell cultures of control (CT) and knockout mice (KO) is plotted as percentage of the total population.
(1.43 MB TIF)

Figure S4 Time course imaging for growth of keratinocytes plated from control and selenoprotein deficient mice. Keratinocytes from control and knockout mice were allowed to attach for 2 h, following which the media was changed and their growth was recorded at regular intervals. A representative image of cells at indicated time-points following attachment.
(2.29 MB TIF)

Figure S5 Time course of appearance of infiltrating macrophages in knockout mice skin section. Infiltrating macrophages in back skin sections of 6, 7, 8, 9 and 16 day old control and knockout mice were stained with F4/80 (macrophage/monocyte specific antigen). Macrophages were noticed around damaged hair follicles. Scale bar: 100 µm.
(4.50 MB TIF)

Figure S6 Expression of granulocytes in knockout mice skin section. Back skin sections from 6, 7, 8, 9 and 16 day old control and knockout mice were stained with myeloperoxidase to determine the presence of granulocytes. Scale bar: 100 µm.
(4.86 MB TIF)

Figure S7 (A) Back skin sections from 10 day old control and knockout mice were stained for integrin beta 1. (B) Expression of integrin beta 1 in freshly isolated keratinocytes from new born control (CT) and knockout (KO) mice was detected by western blotting with beta tubulin as the loading control. Scale bar: 100 µm.
(2.20 MB TIF)

Table S1 Primers used for genotyping PCR.
(0.03 MB DOC)

Table S2 Primer sets used for qPCR.
(0.04 MB DOC)
