# Supplementary figures and images for: Selenoproteins Are Essential for Proper Keratinocyte Function and Skin Development
Source: PLoS One. 2010 Aug 18;5(8):e12249. doi: 10.1371/journal.pone.0012249 (PMC2923614; doi:10.1371/journal.pone.0012249)

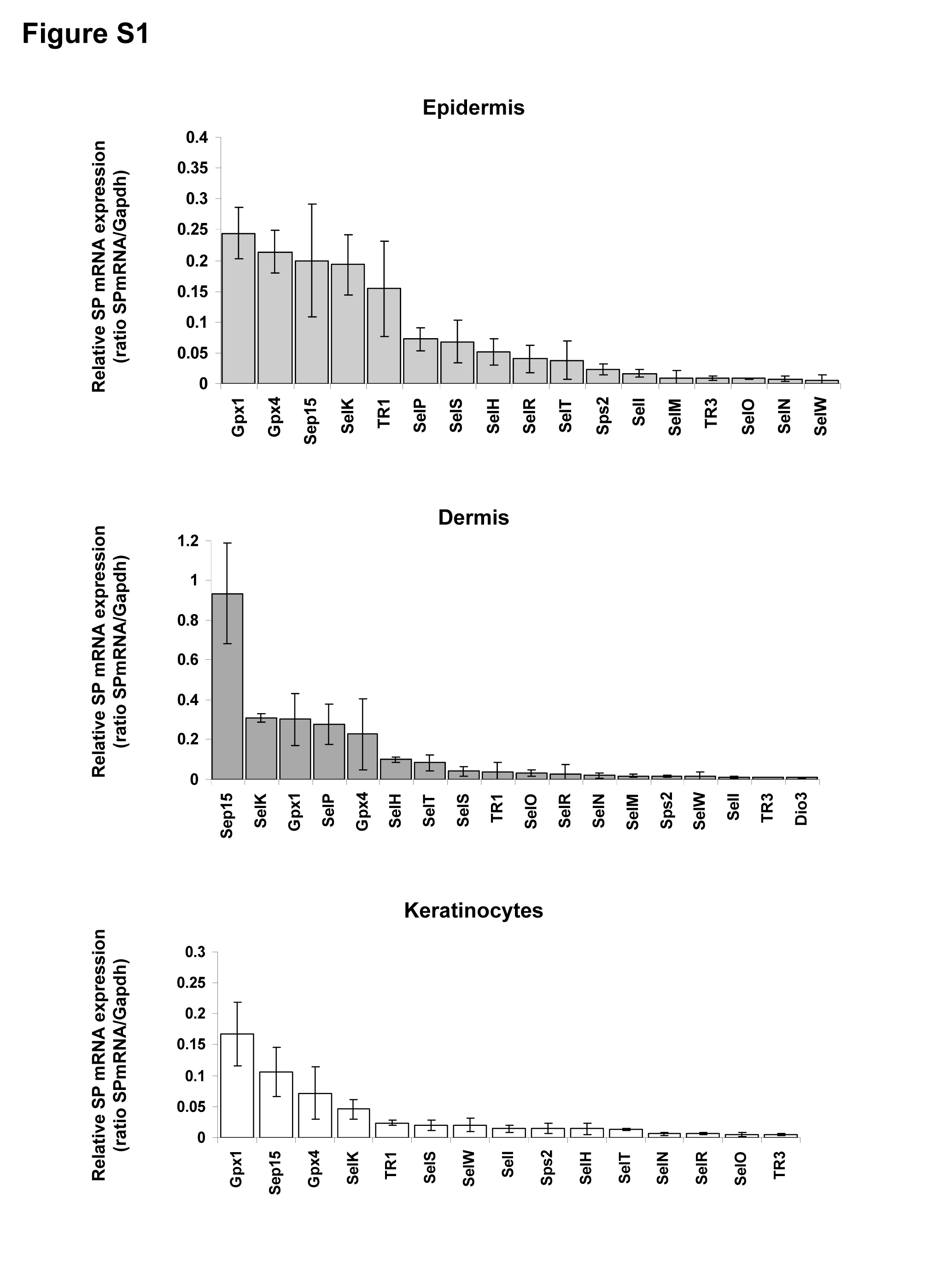

Supplement: Figure S1 — Selective expression of selenoproteins in skin and in cultured keratinocytes. Expression profile of selenoprotein (SP) mRNAs in epidermis and dermis from 6–7 day old mice and primary keratinocytes (cultured for 3 days) was determined by qPCR. The expression level of each selenoprotein was plotted relative to Gapdh. Bars represent the mean values ± S.D for 3 independent experiments. (0.23 MB TIF) [file pone.0012249.s001.tif]

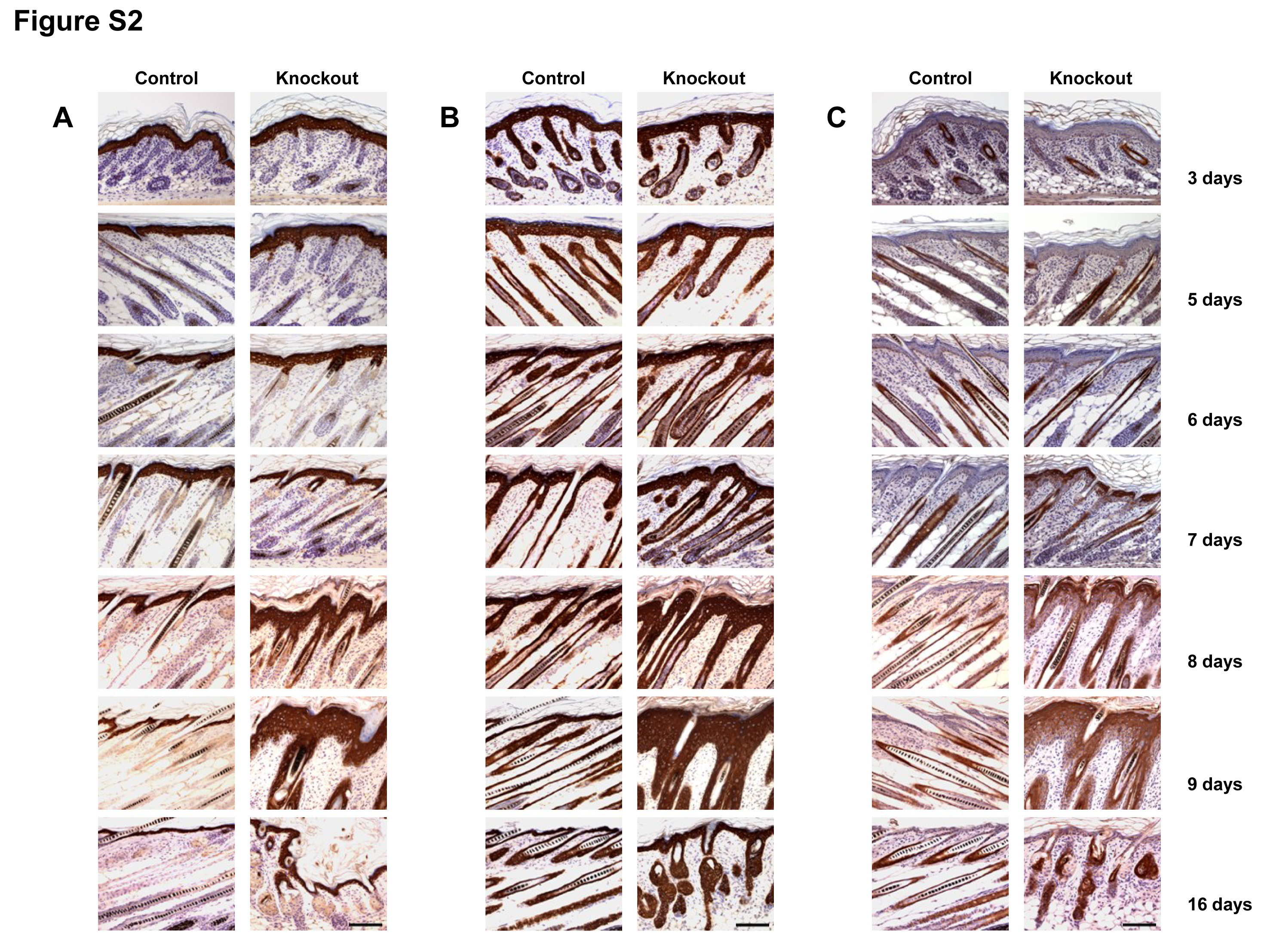

Supplement: Figure S2 — Time course analysis of keratinocyte differentiation markers. Histochemical detection of keratinocyte differentiation markers in back skin sections of 3, 5, 6, 7, 8, 9 and 16 days old mice. (A) Keratin-1 is detected in the suprabasal layer of control and knockout mice and some hair follicles of knockout mice beyond 7 days of age. (B) Keratin-14 is uniformly detected in the ORS of hair follicles and epidermis of the examined control and knockout mouse. (C) Keratin-6 is expressed in suprabasal layer of epidermis of 7, 8 and 9 day old skin sections from knockout mice in addition to its localization in ORS of hair follicles. Scale bar: 100 µm. (7.02 MB TIF) [file pone.0012249.s002.tif]

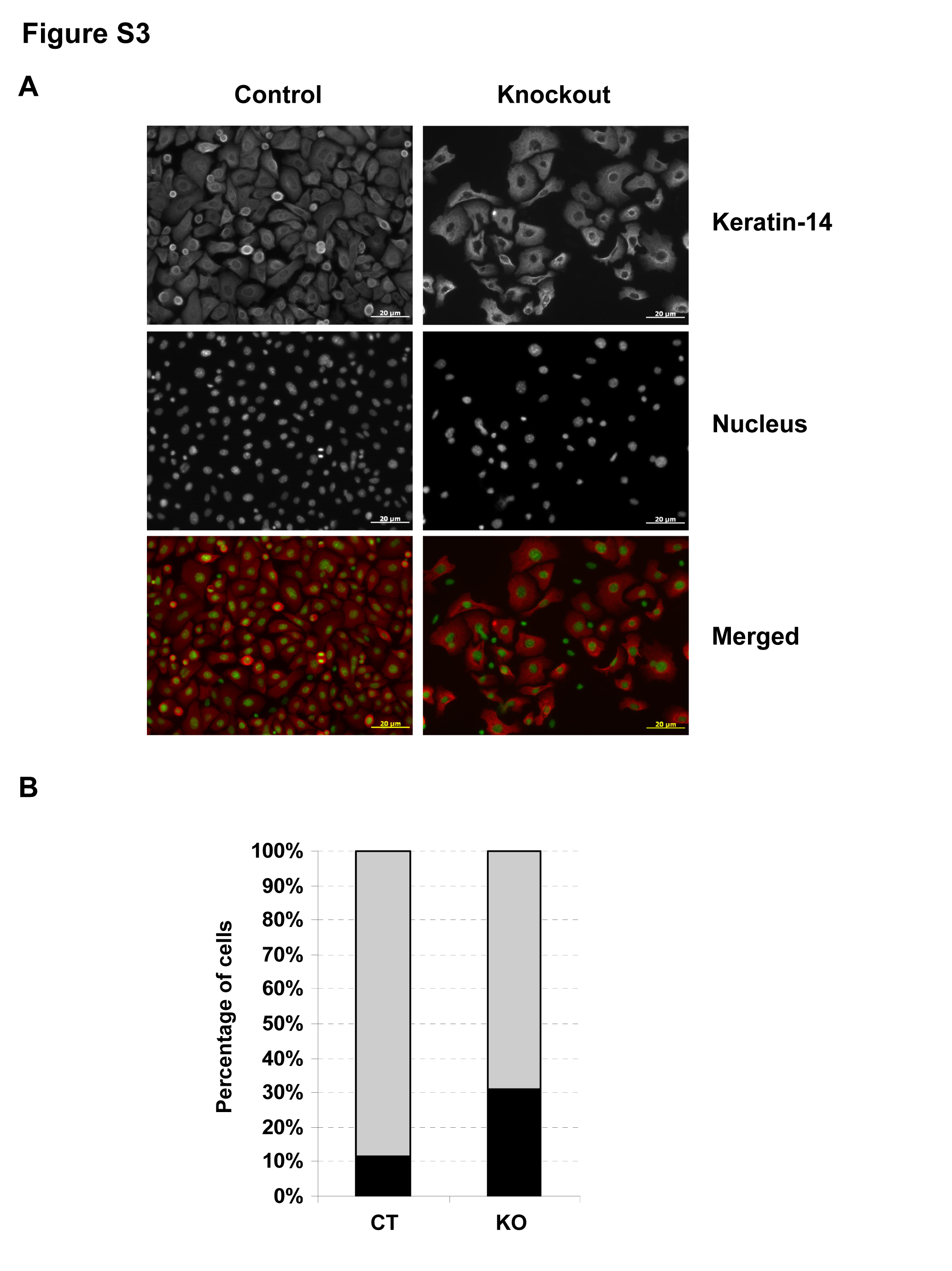

Supplement: Figure S3 — Determination of non-keratinocytic cells in primary keratinocyte preparation. (A) Immunofluorescence staining for keratin-14 in primary keratinocyte preparation from epidermal cell fraction of control and knockout mice, cultured for 3 days. Size bar: 20 µm. (B) The abundance of non-keratinocytic cells (depicted in black) in primary epidermal cell cultures of control (CT) and knockout mice (KO) is plotted as percentage of the total population. (1.43 MB TIF) [file pone.0012249.s003.tif]

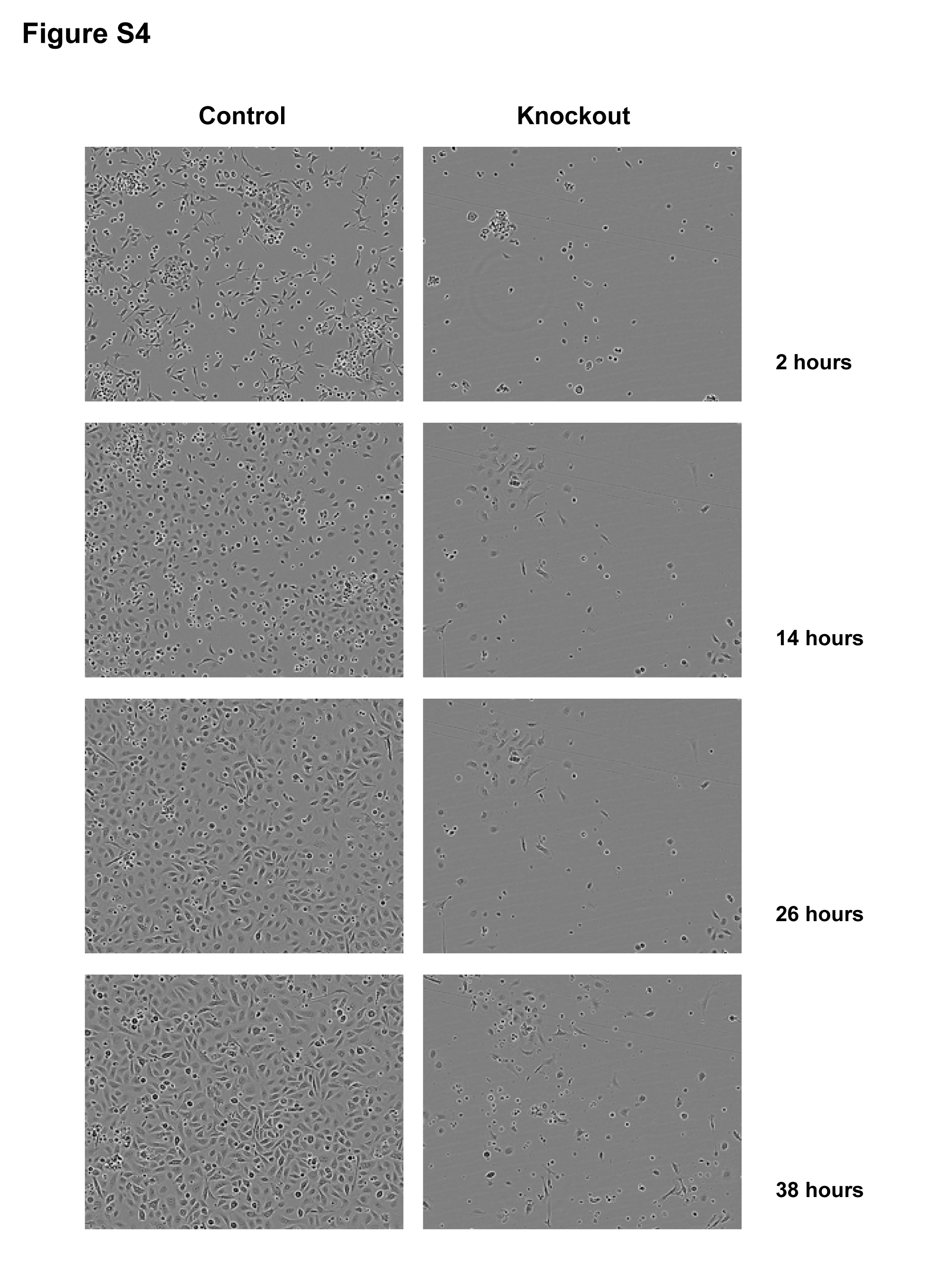

Supplement: Figure S4 — Time course imaging for growth of keratinocytes plated from control and selenoprotein deficient mice. Keratinocytes from control and knockout mice were allowed to attach for 2 h, following which the media was changed and their growth was recorded at regular intervals. A representative image of cells at indicated time-points following attachment. (2.29 MB TIF) [file pone.0012249.s004.tif]

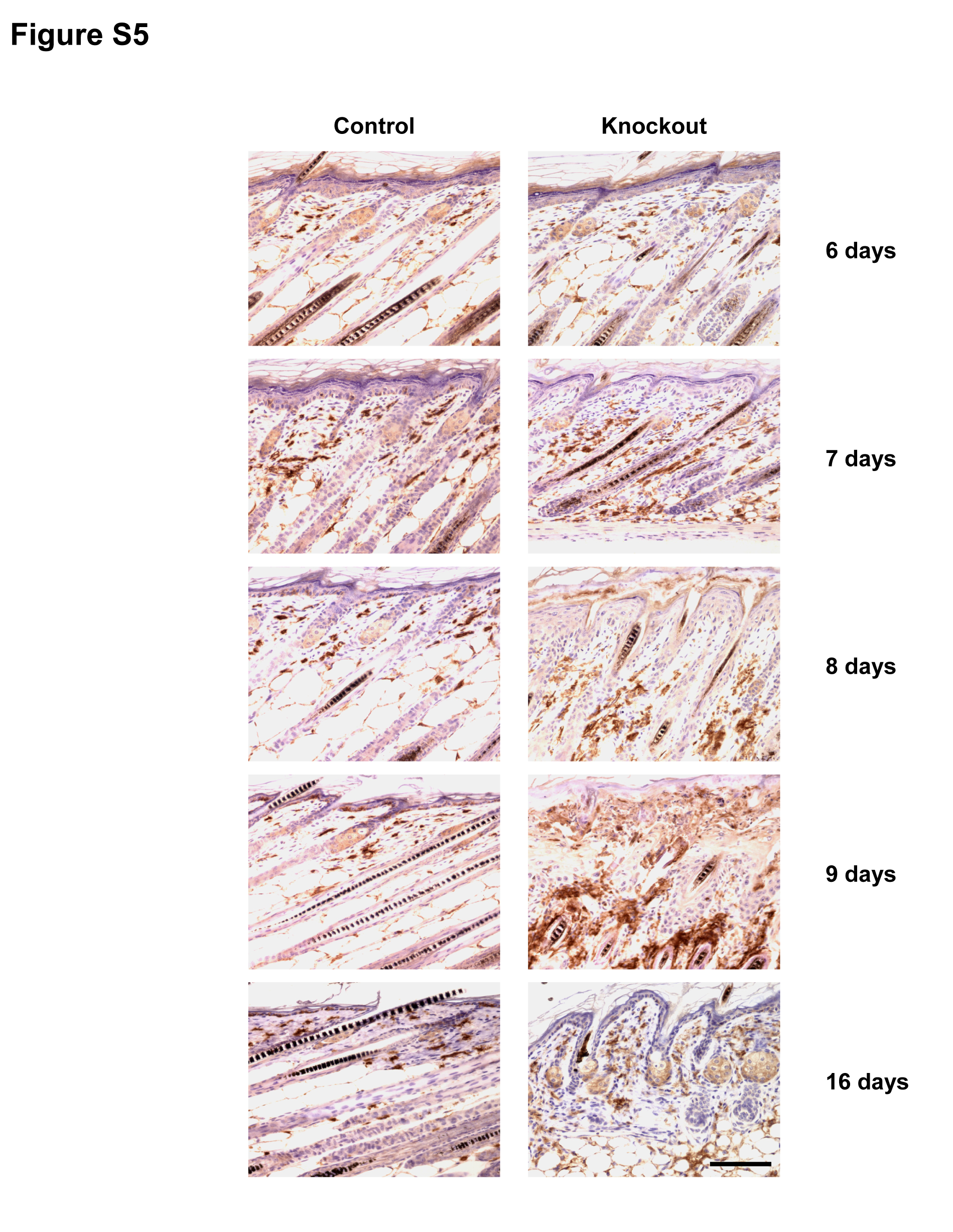

Supplement: Figure S5 — Time course of appearance of infiltrating macrophages in knockout mice skin section. Infiltrating macrophages in back skin sections of 6, 7, 8, 9 and 16 day old control and knockout mice were stained with F4/80 (macrophage/monocyte specific antigen). Macrophages were noticed around damaged hair follicles. Scale bar: 100 µm. (4.50 MB TIF) [file pone.0012249.s005.tif]

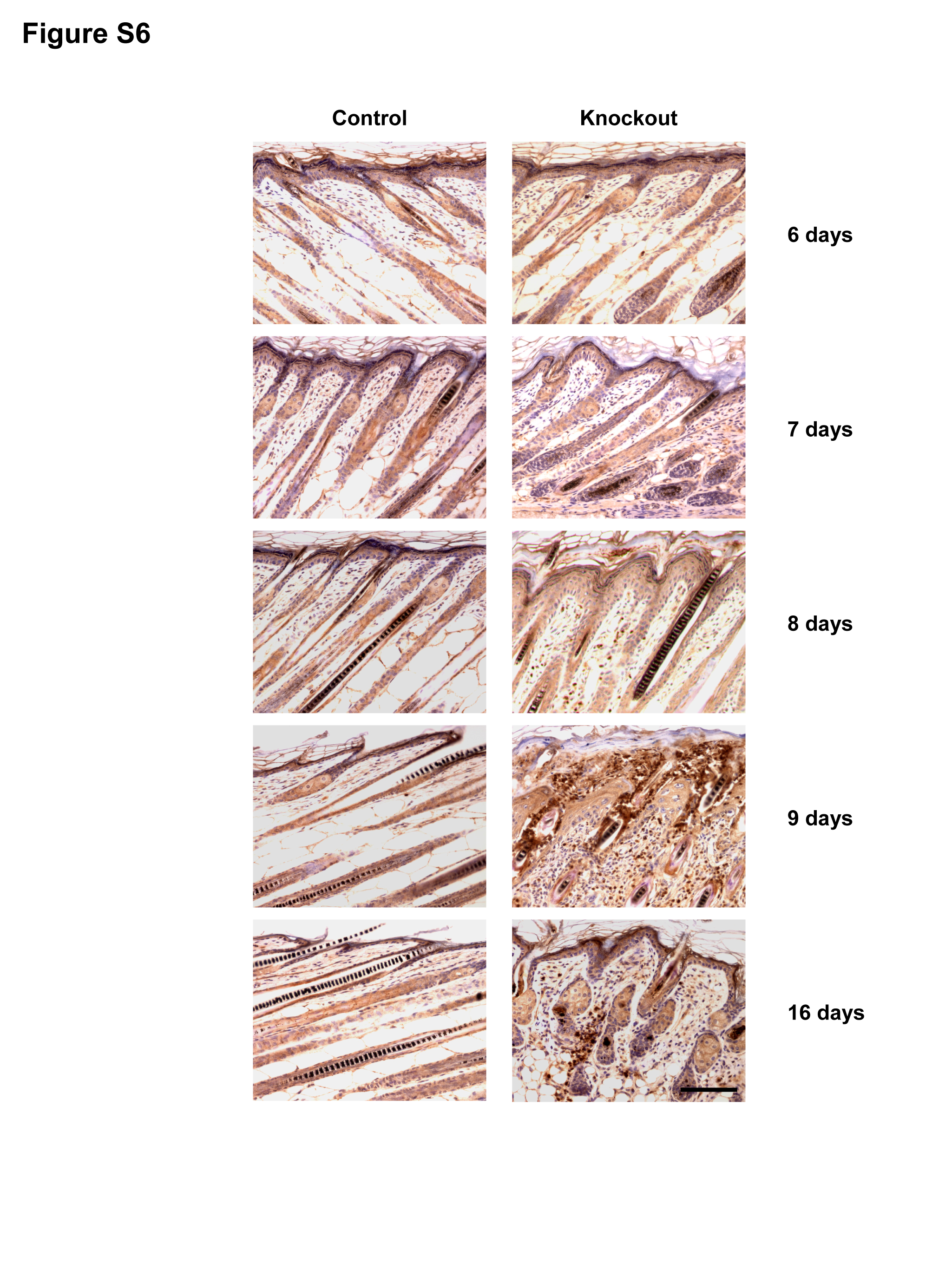

Supplement: Figure S6 — Expression of granulocytes in knockout mice skin section. Back skin sections from 6, 7, 8, 9 and 16 day old control and knockout mice were stained with myeloperoxidase to determine the presence of granulocytes. Scale bar: 100 µm. (4.86 MB TIF) [file pone.0012249.s006.tif]

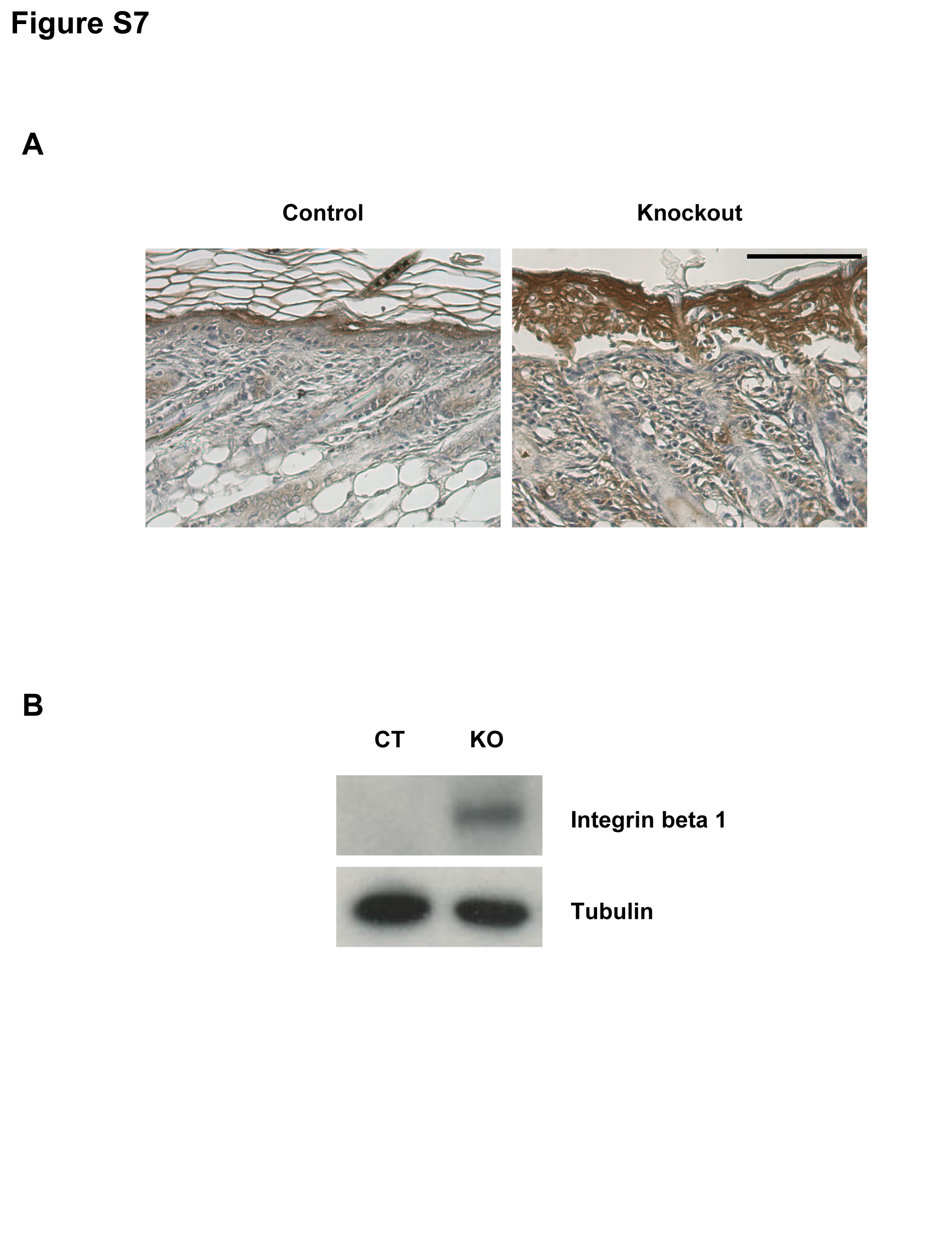

Supplement: Figure S7 — (A) Back skin sections from 10 day old control and knockout mice were stained for integrin beta 1. (B) Expression of integrin beta 1 in freshly isolated keratinocytes from new born control (CT) and knockout (KO) mice was detected by western blotting with beta tubulin as the loading control. Scale bar: 100 µm. (2.20 MB TIF) [file pone.0012249.s007.tif]
