# Supplementary material for: Selenoproteins Are Essential for Proper Keratinocyte Function and Skin Development
Source: PLoS One. 2010 Aug 18;5(8):e12249. doi: 10.1371/journal.pone.0012249 (PMC2923614; doi:10.1371/journal.pone.0012249)
Supplement: Table S1 — Primers used for genotyping PCR. (0.03 MB DOC) [file pone.0012249.s008.doc]

**Table S1.** Primers used for genotyping PCR

| **Primer name** | **Primer Sequence** |
| --- | --- |
|  | |
| CKNO2 | 5’-GCAACGGCAGGTGTCGCTCTGCG-3’ |
| 8RP | 5’-CGTGCTCTCTCCACTGGCTCA-3’ |
| Cre-Forward | 5’-GCTGTTTCACTGGTTATGCGG-3’ |
| Cre-Reverse | 5’-TTGCCCCTGTTTCACTATCCAG-3’ |
