# Supplementary material for: Selenoproteins Are Essential for Proper Keratinocyte Function and Skin Development
Source: PLoS One. 2010 Aug 18;5(8):e12249. doi: 10.1371/journal.pone.0012249 (PMC2923614; doi:10.1371/journal.pone.0012249)
Supplement: Table S2 — Primer sets used for qPCR. (0.04 MB DOC) [file pone.0012249.s009.doc]

**Table S2.** Primer sets used for qPCR

**Gene Accession No Primer Sequence __________**

**Dio1** NM_007860 For AGGAACCATAGGCATTGGAA

Rev CCAGAGAGCCAGATTCCTGT

**Dio2** NM_010050 For GACTCACCAGCCCATGTAAC

Rev CGTGTGACTACATCCAACCA

**Dio3** NM_172119 For CAGAGTGGCACCATCATGTA

Rev TCAGTCACTTGTCCCTTGGT

**Gapdh**  NM_008084 For ATGTGTCCGTCGTGGATCT

Rev GTTGAAGTCGCAGGAGACAA

**Gpx1** NM_008160 For CAGGAGAATGGCAAGAATGA

Rev GAAGGTAAAGAGCGGGTGAG

**Gpx2** NM_030677 For ATCAAACGGCTCCTCAAAGT

Rev GGGACGATATTCAGGGAATG

**Gpx3** NM_008161 For CCCTTAGTGCATTCAGGCTT

Rev GACCTTTACTGGGCAGATGG

**Gpx4** NM_008162 For GCAGGAGCCAGGAAGTAATC

Rev GGCTGGACTTTCATCCATTT

**SelH** BC056177 For CTACCTGTGCAAGTGAACCC

Rev TGAGGCTCAGGAAATTTGAG

**SelI** NM_027652 For CCGTGTTTGCTCTTCACTTT

Rev GCTGACATGTGATATTGGCA

**SelK** NM_019979 For TGGTGGATGAGGAAGGTAAA

Rev ACAGAGCAATCCTTGTTTGG

**SelM** NM_053267 For GATTGGAACCGTCTTCGAG

Rev GTGCTTCATCACCAGGTTGT

**SelO** AK005048 For ATCGACTATGGACCCTTTGG

Rev TTTCTGCAGATTCCACTTGC

**SelR**  NM_013759 For TCCAGTCACTCGAAGTACGC

Rev CTTGCCACAGGACACCTTTA

**SelS** BC011091 For CTTTGCGAGGAGGTGGTTAT

Rev CCTTGCTAATGTCAGAGCGA

**SelT** BC038867 For TGGTCTAAGCTGGAATCTGG

Rev TTTCGGTGCTGATAGGTAGG

**SelV** BC089491 For AATTCCCAAACCTTCTGGAG

Rev GACTCATCCACAAAGCCATC

**Sep15** NM_053102 For TGGAACACAGACAGTGTGGA

Rev TGACCAATGTAAGCATGCAA

**Sps2**  NM_009266 For GATAGTGCCGTGGTAGGAGA

Rev CTCTGGAAACCACCATCTTG

**SelN**  NM_029100 For CCCTAGGTAGCCTTCTGTGC

Rev ACAGGCTGAAGGGTAGCAGT

**SelP** NM_009155 For ATCTTGGCAGCAGTAAGCCT

Rev TCACTTGCTGTGGTGTCTCA

**SelW**  NM_009156 For TAGAGGCAGGGTCCTGAAAG

Rev AATCCATCTCTGGCCTGACT

**TR1**  NM_015762 For CTACAGACCATTGCCTTGCT

Rev ACCTCCTACCCACAAGATCC

**TR2** NM_013711 For TCACTGGAATTGGACTGGAT

Rev ACACAGCCTTTCAGGAACTG

**TR3** NM_153162 For CTGGAATATGGCTGTTGTGG

Rev AGGTGTTGTTGTCTCTGCCA
